# Exercise and nutrition as epigenetic regulators of gene expression: an exploratory scoping review with bibliometric analysis

**DOI:** 10.3389/fnut.2026.1773920

**Published:** 2026-03-10

**Authors:** Hao Zhang, Ruida Yu, Shengrui Cao, Xiaoyang Liu, Cheng Chen, Fei Peng, Yufei Qi

**Affiliations:** 1 Department of Physical Education and Research, Central South University, Changsha, China; 2 Graduate School, Harbin Sport University, Harbin, China; 3 College of Teacher Education, Capital Normal University, Beijing, China; 4 Department of Health and Physical Education, The Education University of Hong Kong, Hong Kong, China

**Keywords:** bibliometric analysis, DNA methylation, epigenetics, exercise, non-coding RNA, nutrition, scope review

## Abstract

**Background:**

Epigenetic processes are increasingly discussed as a potential interface between environmental exposures and genomic function. However, systematic integration and forward-looking synthesis of how exercise and nutrition, considered modifiable lifestyle interventions, are studied in relation to epigenetic contexts and associated health outcomes remains limited.

**Objective:**

This study maps and summarizes the scope of existing evidence on combined interventions of exercise and nutrition in relation to epigenetic contexts and associated health outcomes using a scoping review and bibliometric analysis. It identifies research hotspots and knowledge gaps and characterizes the developmental trajectory of the field, with particular emphasis on its current exploratory stage and future research directions.

**Methods:**

This scoping review followed the five-step framework proposed by Arksey and O’Malley. A systematic search was conducted in PubMed, Web of Science, Scopus, EMBASE, and MEDLINE from database inception to 5 October 2025. Visualization analyses and strategic coordinate modeling were performed using CiteSpace, VOSviewer, and the R bibliometrix package to examine collaboration networks, thematic clusters, and research frontiers across countries, institutions, authors, and keywords.

**Results:**

Seventeen studies involving 1,568 participants were included, with randomized controlled trials as the predominant design (13 studies, 76.5%). The findings indicate that combined interventions of exercise and nutrition are associated with multi-layered epigenetic changes within specific tissues and study contexts, with patterns suggestive of potential synergistic or additive interactions involving DNA methylation, histone modifications, and non-coding RNAs. These epigenetic observations have been reported alongside improvements in metabolic health, enhanced muscular adaptability, modulation of inflammatory processes, and markers related to aging-associated pathways. Bibliometric analysis indicated that publication output remains geographically concentrated, while keyword clustering revealed three core thematic dimensions: intervention strategies, mechanistic exploration, and health outcomes. Keywords with high betweenness centrality included “exercise,” “inflammation,” and “DNA methylation,” whereas “tissue-specific responses” and “epigenetic clocks” appeared as emerging areas of research interest.

**Conclusion:**

Combined interventions of exercise and nutrition demonstrate promising but predominantly exploratory associations within epigenetic contexts and related health outcomes. Current evidence remains limited in scale and heterogeneous in design. Future research may benefit from standardizing epigenetic assessments, strengthening longitudinal and multi-tissue investigations, integrating multi-omics approaches, and enhancing international collaboration. Collectively, the available literature lays conceptual and methodological groundwork for future hypothesis-driven and validation-focused research, rather than supporting validated predictive or precision-oriented applications at present.

## 1 Introduction

Epigenetics the study of mechanisms that induce heritable changes in gene expression or phenotype without altering DNA sequences. Unlike traditional genetic variation, which arises from the addition, deletion, or substitution of base pairs, epigenetic regulation involves the attachment of chemical “marks” to the genome. Epigenetic modifications are fundamentally dynamic and reversible. Their establishment and maintenance are strongly influenced by three main factors: (1) environmental exposures, such as exogenous toxins, pollutants, or pathogens (1, 2); (2) psychosocial stressors, including chronic stress, socioeconomic conditions, and traumatic events (e.g., war or natural disasters) (3); (3) individual lifestyle and physiological states, such as diet, physical activity, sleep patterns, and overall health (4). Despite their high plasticity, epigenetic modifications can be stably transmitted during cell division. This transmission establishes causal links between transient or persistent environmental stimuli and heritable physiological phenotypes.

The core network of epigenetic regulation consists of three major interacting mechanisms: DNA methylation, histone modifications, and non-coding RNAs (5). These mechanisms, acting either synergistically or independently, precisely regulate the spatiotemporal specificity of gene expression. They play pivotal roles in biological processes, including cell differentiation, organismal development, and disease initiation. DNA methylation, the earliest identified and most well-characterized epigenetic modification, involves a covalent modification of cytosine, primarily at CpG dinucleotides (6). Under physiological conditions, CpG islands usually show low methylation and are enriched in gene promoter regions, repetitive sequences, and inactive X chromosome regions (7–9). Aberrant hypermethylation of CpG islands in promoter regions silences gene transcription through two mechanisms: restricting transcription complex binding and recruiting methyl-binding proteins (e.g., MBD family proteins) to compact chromatin structure (9, 10). Conversely, genomic hypomethylation is generally associated with transcriptional activation (8, 9). This mechanism is especially prominent in tumors: hypermethylation of tumor suppressor gene promoters leads to their inactivation, whereas hypomethylation of proto-oncogenes promotes their overexpression (11, 12). For example, in colorectal cancer, the p16INK4a tumor suppressor gene loses function due to aberrant hypermethylation of its promoter. This disrupts cell cycle regulation, ultimately promoting malignant tumor proliferation (13).

Histone modifications are a key component of chromatin remodeling, including nucleosome remodeling, and confer dynamic plasticity to gene expression regulation (5). In eukaryotic cells, DNA wraps around histone octamers (H2A, H2B, H3, H4) to form nucleosomes. The N-terminal tails of these histones undergo various covalent modifications, including methylation and acetylation. These modifications, and their combinations, constitute the “histone code”. Recognized by specific reader proteins, these modifications can directly alter chromatin conformation or recruit remodeling complexes. This facilitates chromatin state transitions, mainly the switching between euchromatin and facultative heterochromatin, whereas conversion from structural heterochromatin to euchromatin is exceptionally rare. Through these processes, histone modifications precisely orchestrate gene expression (14).

Non-coding RNAs (ncRNAs), key components of epigenetic regulation, do not encode proteins but function as molecular scaffolds or guides to precisely regulate gene expression at the transcriptional, chromatin remodeling, and post-transcriptional levels (5). Based on length and functional characteristics, ncRNAs are mainly classified into small interfering non-coding RNAs (e.g., microRNAs and siRNAs) and long non-coding RNAs (lncRNAs). MicroRNAs (miRNAs) mediate post-transcriptional fine-tuning by binding target mRNAs to induce degradation or translational repression. Conversely, siRNAs guide DNA methylation and histone modifications to establish stable transcriptional silencing (15). LncRNAs exhibit greater functional diversity, regulating chromatin structure and function, influencing gene transcription and RNA processing, and playing key roles in gene silencing, activation, and enhancer regulation. For example, enhancer-derived RNAs (eRNAs) promote chromatin looping, facilitate enhancer-promoter interactions, and recruit the transcription machinery to activate gene expression (16). NcRNAs are not mere by-products of genomic transcription. Rather, they interact with mechanisms such as DNA methylation and histone modifications, collectively forming a dynamic, interconnected epigenetic network that achieves precise spatiotemporal regulation of gene expression (5).

Beyond core regulatory mechanisms, environmental and behavioral factors continuously shape the individual epigenome, representing an important external dimension of epigenetic regulation (17). Regular exercise can induce beneficial DNA methylation remodeling, especially in genes associated with oxidative stress, inflammatory responses, and energy metabolism. This improves metabolic health, lowers the risk of chronic diseases, and may delay the aging process (18, 19). Conversely, adverse habits, including prolonged sedentary behavior, smoking, and excessive alcohol consumption, are associated with the accumulation of disease-related epigenetic abnormalities (20). Specific dietary patterns (e.g., Mediterranean and ketogenic diets) and nutrients (e.g., folate, polyphenols, probiotics) modulate gene activity related to cognition, memory, and metabolism through DNA methylation, histone modifications, and ncRNA expression (21–23). Adequate nutrition supports a healthy epigenetic landscape, whereas nutritional deficiencies increase disease susceptibility. Collectively, these findings indicate that both physical activity and nutritional interventions act as effective epigenetic regulators, providing a molecular basis for disease prevention and health promotion.

**FIGURE 1:**
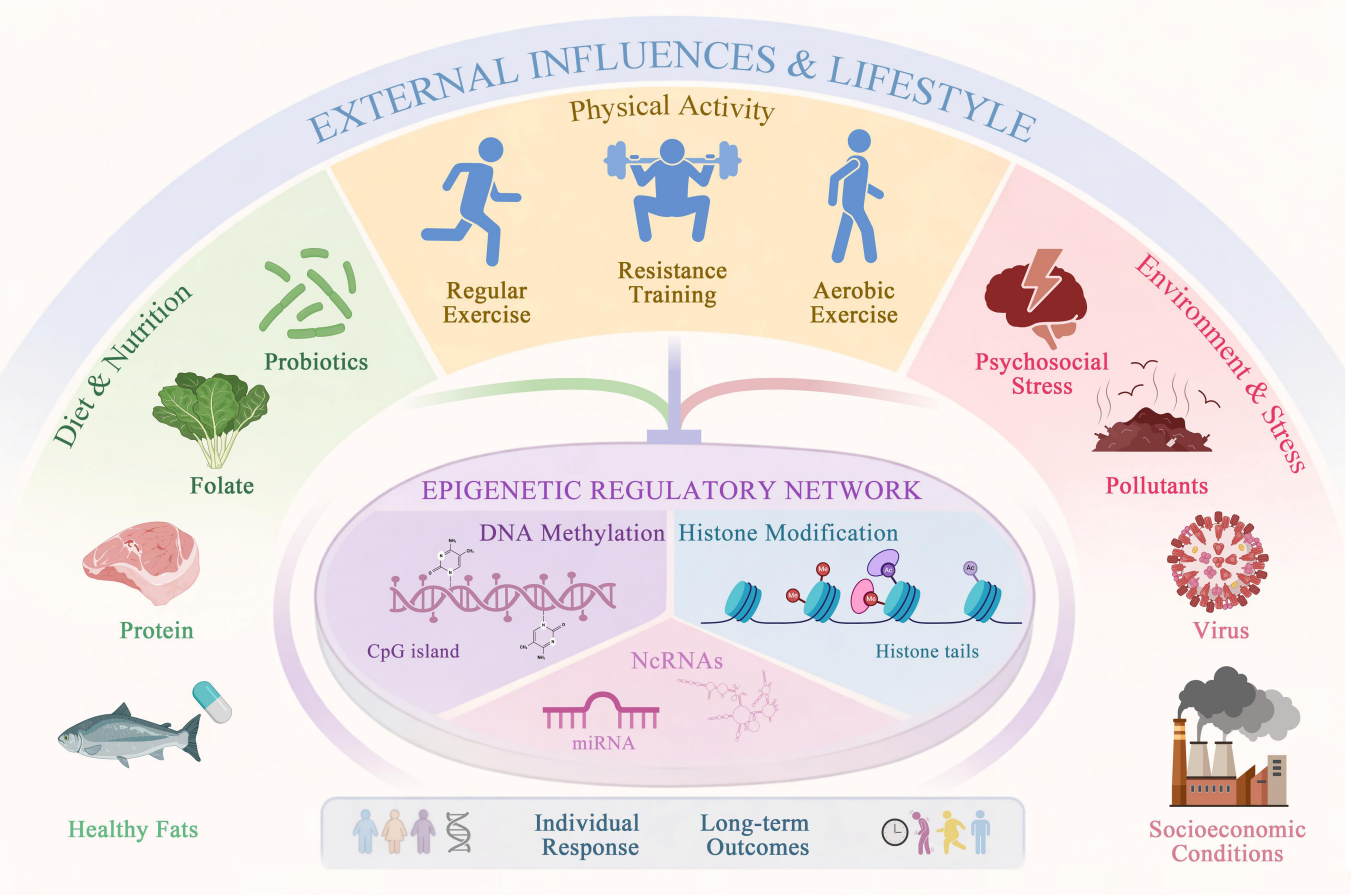
Environmental and lifestyle factors in relation to epigenetic contexts and individual variability.

Individual variability in response to exercise and nutritional interventions is common in health promotion and represents a challenge for personalized and precision-oriented health research. Traditional genetic factors explain only part of this heterogeneity. Epigenetic processes are often discussed as an interface between environmental influences and genomic function, partly because of their dynamic and potentially reversible characteristics. This perspective provides a framework for exploring how lifestyle factors may be biologically embedded, rather than serving as validated determinants of individual outcomes. It also supports further investigation of how exercise and nutrition may relate to physiological adaptation and longer-term health trajectories (Figure 1). Therefore, examining combined interventions within epigenetic contexts holds scientific relevance. However, the current literature remains predominantly exploratory and hypothesis-generating. Rather than supporting predictive or precision applications at present, existing evidence primarily lays conceptual and methodological groundwork for future validation-focused research and evidence-informed lifestyle strategies.

## 2 Materials and methods

### 2.1 Design

This study adopted the scoping review framework proposed by Arksey and O’Malley (24) and was conducted and reported in strict accordance with the PRISMA-ScR (Preferred Reporting Items for Systematic Reviews and Meta-Analyses extension for Scoping Reviews) guidelines (see Supplementary Appendix 1 for details) (25). We searched five core databases (PubMed, Web of Science, Scopus, EMBASE, and MEDLINE), and performed a tiered screening of titles, abstracts, and full texts. Selected studies were exported into standardized text formats and subjected to bibliometric and visualization analyses using CiteSpace, VOSviewer, and the R bibliometrix package. Co-author network analysis was conducted to map collaboration networks among countries, institutions, and authors in the field of “Combined interventions of exercise and nutrition in relation to epigenetic contexts and associated health outcomes” providing an overview of current research collaboration. Clustering, strategic coordinate analysis, and keyword overlay visualization techniques were applied to identify distinct themes and reveal emerging research hotspots.

### 2.2 Rationale for the scope review

Unlike systematic reviews and narrative syntheses, scoping review methodologies are designed to systematically map the research landscape within a specific disciplinary field, including its publication volume and core characteristics. Rather than conducting evidence quality assessments or synthesizing effect sizes of existing studies (26), these methodologies clarify core disciplinary concepts, identify key knowledge gaps, and establish a foundational framework for subsequent research endeavors. In this study, we aim to elucidate the intrinsic structural features of the relevant literature by systematically mapping research activities in the target field, and further facilitate cross-disciplinary knowledge integration and dissemination. Accordingly, our primary focus is on delineating the current state, thematic distribution, and developmental trajectory of research on “Combined interventions of exercise and nutrition in relation to epigenetic contexts and associated health outcomes”. Therefore, the adoption of the scoping review methodology is fully justified and methodologically appropriate.

### 2.3 Research questions and conceptual framework

This scoping review seeks to address three core questions: (1) Which epigenetic patterns or regulatory themes have been reported in this research domain, and how have existing studies described associations between combined interventions of exercise and nutrition, epigenetic contexts, and related health outcomes? (2) What methodological characteristics and major research gaps are evident in the current evidence concerning population types, intervention protocols, and outcome measures? (3) Which emerging directions may warrant prioritization in future research to advance conceptual understanding and inform clinical and public health inquiry?

To ensure conceptual clarity and consistency, key terms were defined *a priori*. In this scoping review, the term “synergy” is used descriptively to refer to reported patterns of comparative association observed in studies of combined interventions of exercise and nutrition. Specifically, it denotes situations in which the combined intervention is associated with epigenetic or health-related changes that may be greater than those observed with either component alone, when such comparative evidence is explicitly reported in the original studies. When comparator groups are absent, findings are described using more cautious terms such as “combined,” “concurrent,” or “potentially additive” rather than “synergistic.” This definition reflects the descriptive and mapping nature of scoping reviews and avoids causal inference.

### 2.4 Literature search strategy

To comprehensively identify relevant literature, we systematically searched five databases: PubMed, Web of Science, Scopus, EMBASE, and MEDLINE. The search period extended from the inception of each database to 5 October 2025. The search strategy combined free-text terms and subject headings, employing Boolean operators without geographical restrictions. In addition to electronic searches, references from included studies were manually backtracked to supplement relevant research. Only English-language publications were included, and detailed search strategies for each database are provided in Supplementary Appendix 2.

### 2.5 Literature screening

The Joanna Briggs Institute recommends using the “population, concept, and context” (PCC) framework to define the core elements of scoping reviews (27). Accordingly, this study applied the PCC framework to guide literature screening. The “population” included humans of all ages, encompassing both healthy individuals and patients; the “concept” comprised studies investigating the association between combined interventions of exercise and nutrition and epigenetic markers or epigenetic contexts; and the “context” was unrestricted. Based on this framework, the following inclusion and exclusion criteria were established: (1) Inclusion criteria: ➀ Original studies, including cross-sectional, cohort, case-control, and interventional studies (e.g., randomized/non-randomized controlled trials, pilot studies); ➁ Full-text publications; ➂ Studies published from database inception to 5 October 2025. (2) Exclusion criteria: ➁ Non-English publications; ➁ Non-original research types, including reviews, editorials, reports, letters, and conference abstracts; ➂ Animal studies; ➃ Studies with exercise-only or nutrition-only interventions; ➄ Studies using exercise solely as a validation tool rather than an intervention (e.g., examining the effects of creatine intake on exercise performance). Retrieved records were imported into Zotero for deduplication. Subsequently, two researchers (ZH and YRD) independently screened titles and abstracts against the predefined inclusion and exclusion criteria. Articles that passed initial screening proceeded to full-text review. Inter-rater reliability was evaluated using Cohen’s kappa coefficient (Table 1) (28), indicating high agreement (*K* = 0.951). The final set of included studies was determined based on the full-text review. In cases of disagreement, a third researcher (QYF) was consulted to arbitrate and reach consensus.

**TABLE 1:** Kappa consistency between two annotators (ZH **vs.** YRD).

Kappa consistency test				
Pair	Kappa Value	Standard Error	z-Statistic	*P-*value
ZH vs. YRD	0.951	0.014	67.632	0.000***

*** denote significance levels of 1%.

### 2.6 Data extraction and analysis

Two researchers (ZH and YRD) independently extracted data using pre-designed standardized data extraction forms, with subsequent cross-checking. Discrepancies were resolved through joint discussion or consultation with a third researcher (QYF) to achieve consensus. Extracted data primarily included: ➀ Basic study information: authors, publication year, and country; ➁ Study characteristics: study design, research objectives, participant characteristics, and study location; ➂ Intervention and mechanism details: intervention components and types of epigenetic mechanisms; ➃ Outcome measures: categories of health outcomes and corresponding findings. This study strictly adhered to scoping review principles, focusing on describing study characteristics and distribution, without performing evidence quality assessments or synthesizing results.

### 2.7 Bibliometric analysis

CiteSpace, VOSviewer, and the R-based Bibliometrix package are three widely used tools in bibliometric research (29). CiteSpace specializes in constructing knowledge maps and identifying research hotspots, visually representing research structures and developmental dynamics through analyses of publication trends, clusters, and keyword bursts (30). VOSviewer similarly supports the construction of visualized networks, such as collaboration and co-occurrence graphs. It can summarize key information, such as countries, institutions, authors, and journals, while extracting highly co-cited documents and keywords (31). As a specialized R toolkit, Bibliometrix provides a comprehensive overview of a field, evaluates the influence of authors and countries, generates strategic keyword maps, and calculates betweenness centrality, facilitating the exploration of research hotspots and evolutionary trends.

Therefore, to comprehensively analyze collaborative patterns, knowledge structures, and developmental dynamics within this field, this study integrates CiteSpace, VOSviewer, and the R Bibliometrix package to perform bibliometric and visualization analyses. The specific workflow comprised the following steps: ➀ Publication trend analysis: mapping the temporal distribution of literature; ➁ Collaboration network construction: identifying core research groups and international collaboration patterns based on co-authorship relationships at national, institutional, and author levels; ➂ Keyword analysis: identifying research themes and hotspot evolution pathways through co-occurrence networks and tag clouds; ➃ Strategic coordinate and evolutionary analysis: plotting thematic strategic coordinate maps to identify potential future research directions. Prior to analysis, keywords were cleaned and merged for synonyms to remove irrelevant terms, thereby enhancing analytical precision and visualization quality.

## 3 Results

### 3.1 Literature screening process and characteristics of included studies

**FIGURE 2:**
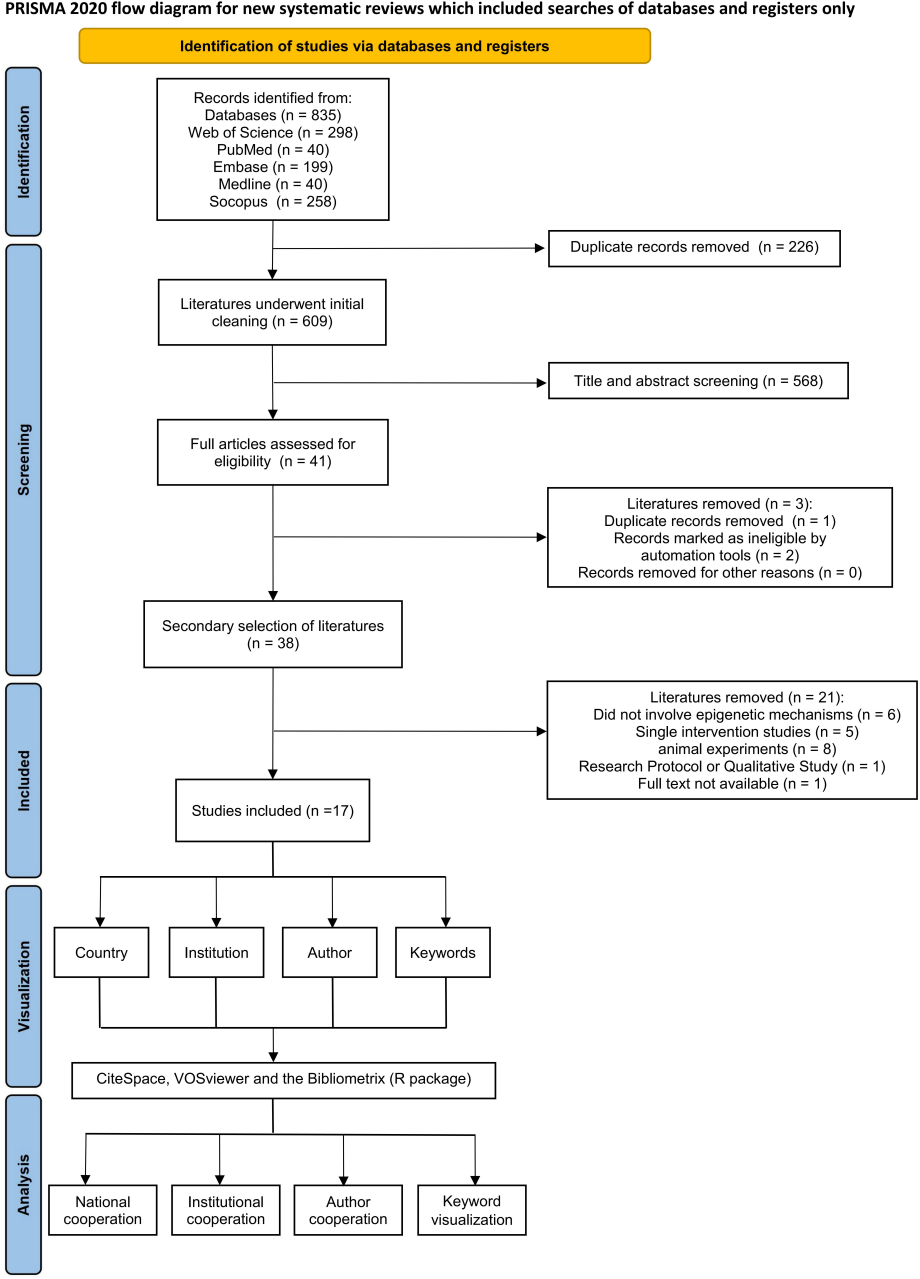
Flow diagram of the article selection process based on PRISMA-ScR guidelines.

This study conducted a comprehensive literature search across five electronic databases, including PubMed, MEDLINE, Web of Science, EMBASE, and Scopus, yielding 795 records. After removal of duplicate entries, 609 unique records were screened. Title and abstract screening excluded 568 records, resulting in 41 full-text articles assessed for eligibility. Following full-text review, 17 studies met the inclusion criteria and were included in this scoping review. The study selection process followed PRISMA-ScR guidelines and is illustrated in Figure 2.

The 17 included studies involved a total of 1,568 participants and employed diverse research designs, including randomized controlled trials (RCTs), crossover studies, and quasi-randomized studies. RCTs represented the predominant design (13 studies, 76.5%) (32–44), alongside three crossover studies (45–47) and one quasi-randomized study (48). Sample sizes ranged from 7 participants (47) to 777 (33), with 11 studies (64.7%) enrolling fewer than 50 participants (34, 37, 39–47), which may reflect the field’s current exploratory stage. Participants represented heterogeneous populations, including healthy adults (32, 44, 47), overweight or obese individuals (35, 41, 48), older adults (33, 37, 38, 40, 43), professional or amateur athletes (34, 42, 45, 46), and individuals with hyperglycemia (38). Intervention durations varied considerably, ranging from acute exercise sessions (40, 42, 44, 47) to programs extending up to 3 years (33). Combined intervention strategies included aerobic and resistance exercise (39, 45), Mediterranean-style dietary patterns (35, 36), and nutritional supplements such as omega-3 fatty acids (33), vitamin D (33, 42), and MitoQ (34).

### 3.2 Epigenetic marker domains and associated health-related patterns

Building on the study characteristics summarized above, the included literature can be descriptively grouped into three primary epigenetic marker domains: DNA methylation (6 studies), histone modifications (2 studies), and ncRNAs (9 studies). These markers were assessed within the populations and tissue or sample sources examined in each study, most commonly blood and/or skeletal muscle. Reported epigenetic measures were described alongside physiological parameters, including body composition, metabolic indicators, inflammatory markers, vascular function, and aging-related metrics. Given the heterogeneity of populations, tissues, and analytical platforms, interpretation beyond the specific study contexts should be undertaken cautiously.

To improve clarity and transparency, Table 2 provides a structured overview of study characteristics, intervention components, epigenetic marker categories, and reported outcomes. Table 3 further stratifies the studies by population type, tissue or sample source, and epigenetic marker category, allowing clearer visualization of how the available evidence is distributed across biological and methodological dimensions. Supplementary Appendix 3 lists the epigenetic markers assessed in each study, while Supplementary Appendix 4 offers brief descriptive summaries of study designs and reported findings to provide additional contextual detail within the scoping framework.

**TABLE 2:** Characteristics of included studies examining combined interventions of exercise and nutrition.

Source	Population	Intervention	Epigenetic mechanism	Outcomes
Hipler et al. (32) (UK^➀^, 2019)	Adults with unhealthy lifestyles	Increased MVPA^➁^+ individualized nutrition counseling	DNA methylation of chronic disease-related genes	Changes in chronic disease risk-related markers
Bischoff-Ferrari et al. (33) (CH^➀^, 2025)	Community-dwelling older adults	Home-based strength training + vitamin D + omega-3	DNA methylation-based epigenetic clock (GrimAge)	Changes in biological aging indicators
Aminzadeh et al. (34) (IR^➀^, 2023)	Professional male athletes	Cycling ergometer training + MitoQ	miRNA regulation related to oxidative stress and vascular inflammation	Changes in vascular function and endurance
Hunter et al. (45) (UK^➀^, 2019)	Healthy male athletes	High-intensity rowing + omega-3 + extra virgin olive oil	DNA methylation of leukocyte metabolic and inflammatory genes	Changes in leukocyte epigenetic profiles
Heianza et al. (35) (US^➀^, 2022)	Abdominally obese adults	Structured physical activity + Mediterranean low-carbohydrate diet	Circulating miR-99/100 family miRNA expression	Changes in visceral fat and glucose-related parameters
Fiorito et al. (36) (IT^➀^, 2021)	Postmenopausal women	Moderate-to-high intensity exercise + Mediterranean diet	DNA methylation-based aging indicators and genome stability	Changes in epigenetic aging metrics
Fiorito et al. (36) (IT^➀^, 2021)	Older women	Physical exercise + grape juice	Histone acetylation	Changes in oxidative stress and inflammatory markers
Aida et al. (38) (JP^➀^, 2025)	Hyperglycemic older adults	Interval walking training + high-polyphenol rice	DNA methylation of NF_*k*_B2^➅^	Changes in glycemic control and inflammatory markers
Schwarz et al. (39) (US^➀^, 2019)	Recreational male athletes	Structured resistance training + complex supplements	Skeletal muscle miRNA expression	Changes in lean mass and strength-related outcomes
D’Souza et al. (40) (NZ^➀^, 2019)	Older men	Resistance exercise + whey protein	Skeletal muscle miRNA expression	Changes in anabolic signaling markers
Parr et al. (41) (AU^➀^, 2016)	Overweight or obese adults	Isocaloric restricted diet + exercise	Circulating miRNA profiles	Changes in weight loss-related responses
Pasusznik-Lewandoska et al. (42) (PL^➀^, 2019)	Marathon runners	Vitamin D supplementation + single 100-km ultramarathon	Inflammation-related gene expression and miRNA profiles	Changes in exercise-induced inflammatory responses
Morikawa et al. (43) (JP^➀^, 2018)	Older women	Interval walking training + post-training soy protein	DNA methylation of NFKB2^➂^	Changes in inflammatory markers
Martins et al. (46) (BR^➀^, 2020)	Adolescent athletes	Regular training + grape juice	Histone H4 acetylation	Changes in oxidative stress and DNA damage markers
Margolis et al. (44) (US^➀^, 2017)	Healthy adults	Variable exercise modes + amino acids or carbohydrates	Skeletal muscle miRNA expression	Changes in protein synthesis-related signaling
Margolis et al. (47) (US^➀^, 2022)	Healthy adults	Aerobic exercise + carbohydrate supplementation	Skeletal muscle miRNA expression	Changes in protein breakdown and recovery-related markers
Tang et al. (48) (CN^➀^, 2019)	Obese male adolescents	Aerobic training + calorie-restricted balanced diet	Serum miR-126 expression	Changes in body composition, glucose metabolism, and endothelial function

➀ Country abbreviations: CH, Switzerland; IR, Iran; US, United States; IL, Israel; IT, Italy; BR, Brazil; JP, Japan; NZ, New Zealand; AU, Australia; PL, Poland; ZA, South Africa; CN, China, ➁ MVPA, moderate-to-vigorous physical activity; ➂ NFKB2 (gene symbol), nuclear factor kappa B subunit 2.

**TABLE 3:** Stratification of included studies by population type, tissue or sample source, and epigenetic marker category.

Study	Population type	Tissue or sample source	Epigenetic marker category
Hibler et al. (32) (UK, 2019)	Adults with unhealthy lifestyles	Blood (leukocytes)	DNA methylation
Bischoff-Ferrari et al. (33) (CH, 2025)	Community-dwelling older adults	Blood	DNA methylation-based epigenetic clock
Aminzadeh et al. (34) (IR, 2023)	Professional male athletes	Plasma	miRNA
Hunter et al. (45) (UK, 2019)	Healthy male athletes	Blood (leukocytes)	DNA methylation
Heianza et al. (35) (US, 2022)	Abdominally obese adults	Plasma	miRNA
Fiorito et al. (36) (IT, 2021)	Healthy postmenopausal women	Blood	DNA methylation-based aging indicators
Fiorito et al. (36) (IT, 2021)	Healthy older women	Skeletal muscle	Histone modification
Aida et al. (38) (JP, 2025)	Hyperglycemic older adults	Blood	DNA methylation
Schwarz et al. (39) (US, 2019)	Recreational male athletes	Skeletal muscle	miRNA
D’Souza et al. (40) (NZ, 2019)	Healthy older men	Skeletal muscle	miRNA
Parr et al. (41) (AU, 2016)	Overweight or obese adults	Blood	miRNA
Pastuszak-Lewandoska et al. (42) (PL, 2020)	Recreational endurance athletes	Blood	Gene expression and miRNA
Morikawa et al. (43) (JP, 2018)	Older women	Blood	DNA methylation
Martins et al. (46) (BR 2020)	Adolescent athletes	Skeletal muscle	Histone modification
Margolis et al. (44) (US, 2017)	Healthy adults	Skeletal muscle	miRNA
Margolis et al. (47) (US, 2022)	Healthy adults	Skeletal muscle	miRNA
Tang et al. (48) (CN, 2019)	Obese male adolescents	Serum	miRNA

DNA methylation, the most extensively investigated epigenetic regulatory mechanism, has been used to characterize genome-wide and gene-specific epigenetic responsiveness to lifestyle interventions. In studies involving metabolically at-risk populations with multiple unhealthy behaviors, combined interventions of exercise and nutrition have been reported to be associated with alterations in genome-wide differentially methylated regions (DMRs), with a predominance of hypomethylated loci. These DMRs were frequently enriched in pathways related to glucose metabolism, energy homeostasis, and cell proliferation, including the PI3K/AKT and Wnt/β-catenin pathways (32). In the context of inflammatory regulation, several studies have observed that exercise combined with specific nutritional components, such as high-pressure processed rice or dried tofu, was associated with concurrent changes in methylation patterns within inflammation-related genes, including the NFKB2 promoter region (38, 43). These methylation changes were reported alongside improvements in glycemic control and inflammatory markers. When such functional foods were incorporated into sustained exercise programs, additional methylation changes in inflammatory-related genes were observed, which may reflect additive or context-dependent associations (i.e., where the observed relationship may vary depending on specific experimental conditions, biological settings, or individual characteristics) rather than definitive mechanistic effects. With respect to aging-related pathways, combined supplementation with omega-3 fatty acids, vitamin D, and exercise has been associated with favorable changes in DNA methylation-based aging indicators, such as PC-PhenoAge and GrimAge, as well as methylation changes in plasma protein–related genes, including PAI-1 and leptin (33). Longer-term interventions have also been reported to coincide with reductions in epigenetic mutation burden and slower changes in epigenetic aging metrics (36). However, these observations are primarily derived from exploratory and context-specific studies, and their long-term biological significance remains to be fully clarified.

Evidence regarding histone modifications remains limited and heterogeneous. Preliminary evidence from a single study suggests that specific nutritional supplements, such as grape juice, may be associated with attenuation of exercise-related changes in histone H4 acetylation (46). In contrast, a separate investigation reported no significant alterations in global H3 or H4 acetylation following combined interventions in healthy older populations (37). Collectively, these inconsistent findings indicate that histone modification responses may be highly dependent on tissue specificity, intervention protocols, and population characteristics. Thus, their functional relevance within combined interventions remains exploratory and necessitates further verification.

ncRNAs, particularly miRNAs, have frequently been examined as rapidly responsive epigenetic signals following combined interventions of exercise and nutrition. At the level of metabolic homeostasis, changes in circulating miR-99/100 family expression, including reported downregulation, have been observed in association with alterations in ectopic fat distribution, glycemic parameters, and markers of β-cell function, although these relationships remain correlational (35). In skeletal muscle, whey protein supplementation following resistance exercise has been associated with changes in the expression of several catabolism-related miRNAs, alongside concurrent alterations in Akt–mTOR signaling activity (40). Similarly, post-aerobic exercise carbohydrate intake has been reported to coincide with altered expression of miRNAs such as let-7i-5p and miR-195-5p, in parallel with changes in muscle catabolic gene expression (47). In vascular and inflammatory contexts, exercise combined with antioxidant or dietary interventions has been associated with shifts in circulating miRNA profiles, including reduced expression of pro-inflammatory miRNAs (e.g., miR-155, miR-19b) and increased expression of miR-146a, alongside concurrent changes in vascular-related outcomes (34). In obese adolescent populations, combined aerobic exercise and dietary restriction has likewise been reported to coincide with changes in serum miR-126 levels, occurring in parallel with weight loss and improvements in microvascular endothelial function (48). Collectively, these findings suggest that miRNAs may serve as responsive epigenetic signals linking combined interventions to metabolic and vascular phenotypes, although their mechanistic or predictive significance remains to be fully established.

Notably, distinct epigenetic mechanisms may not operate in isolation but instead form an interconnected regulatory network. One study reported that acute aerobic exercise combined with n-3 polyunsaturated fatty acids or extra virgin olive oil supplementation was associated with changes in the expression of DNA methyltransferases (DNMT1, DNMT3a, DNMT3b), alongside concomitant methylation changes in genes such as PPARGC1A and IL6 (45); however, these observations do not establish causality. Separately, alterations in specific miRNAs have also been observed in association with anabolic signaling pathways such as Akt–mTOR, suggesting possible feedback relationships within these systems (40). Taken together, these preliminary findings are consistent with the possibility that combined interventions may be associated with coordinated epigenetic responses across multiple levels, rather than acting through singular, isolated mechanisms.

Overall, available evidence suggests that combined exercise and nutrition interventions may be associated with concurrent changes across multiple epigenetic layers, particularly DNA methylation and miRNA profiles. These observations are derived primarily from exploratory studies. The extent to which such multi-layered epigenetic changes correspond to causal mechanisms, durable adaptations, or context-dependent responses remains to be clarified through larger, longitudinal, and multi-tissue investigations. At present, epigenetic markers, including circulating miRNAs and DNA methylation–based aging indicators, are best considered dynamic and responsive signals, rather than validated predictive or precision biomarkers.

### 3.3 A Panorama of bibliometrics: global landscape and developmental trajectories

#### 3.3.1 Publication timelines and domain development trends

**FIGURE 3:**
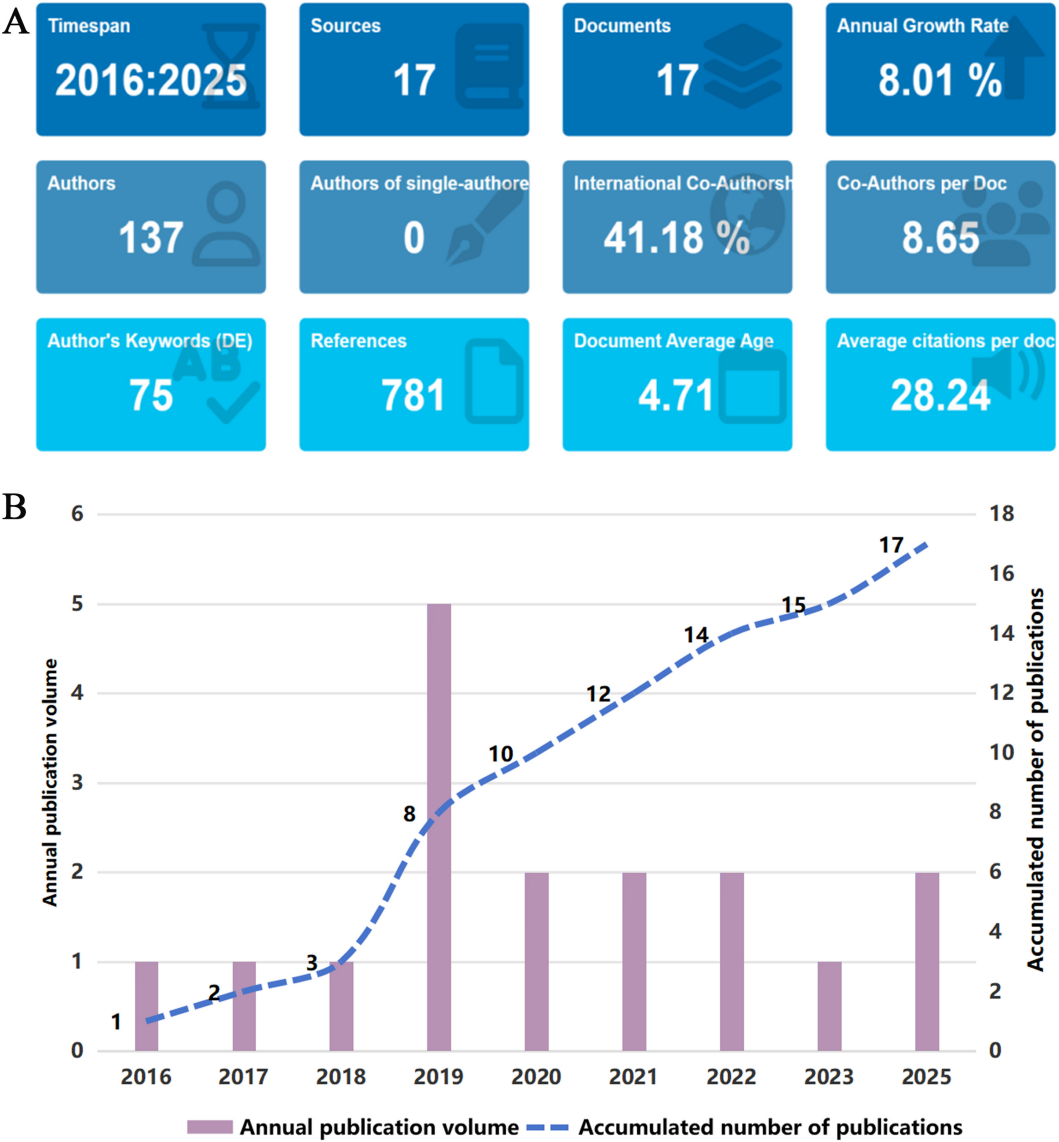
Basic information analysis. **(A)** Abstract information of included studies. **(B)** Annual publication trend chart.

Using CiteSpace and Bibliometrix, we summarized temporal patterns and descriptive characteristics of the included studies to contextualize the developmental status of this research area. Since the first eligible empirical study in 2016 examining combined interventions of exercise and nutrition in relation to epigenetic contexts and associated health outcomes, the field has accumulated 17 eligible empirical studies authored by 137 scholars, generating 781 citations and 75 author keywords (Figure 3A). These indicators suggest a small but emerging body of literature. Annual publication trends (Figure 3B) show a fluctuating upward pattern, with a peak in 2019. Although annual output declined slightly thereafter, the cumulative curve continued to rise, indicating sustained, though modest, growth. Overall, the limited number of empirical studies and their temporal clustering are consistent with the early-stage and exploratory nature of this interdisciplinary domain. Geographically, the United States contributed six studies, China and Italy contributed two each, and additional publications originated from the United Kingdom, Switzerland, New Zealand, Japan, and other regions. The distribution suggests that empirical research remains concentrated within a relatively small group of countries, providing contextual background for interpreting the current scope and representativeness of the available evidence.

#### 3.3.2 Collaborative analysis of countries, institutions and authors

**FIGURE 4:**
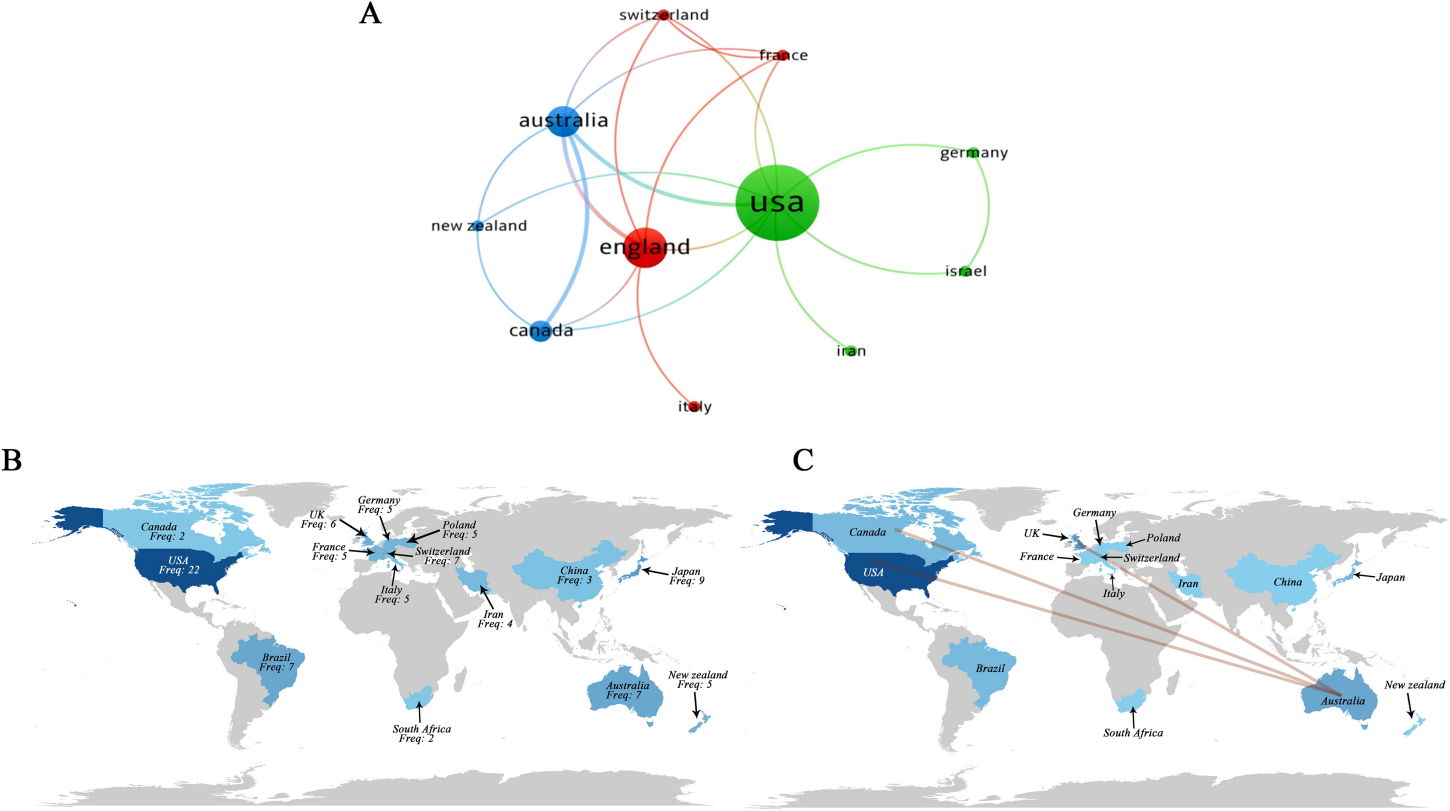
National-level research landscape and collaboration in combined interventions of exercise and nutrition. **(A)** National co-occurrence network. **(B)** Global research distribution. **(C)** International collaboration status.

**FIGURE 5:**
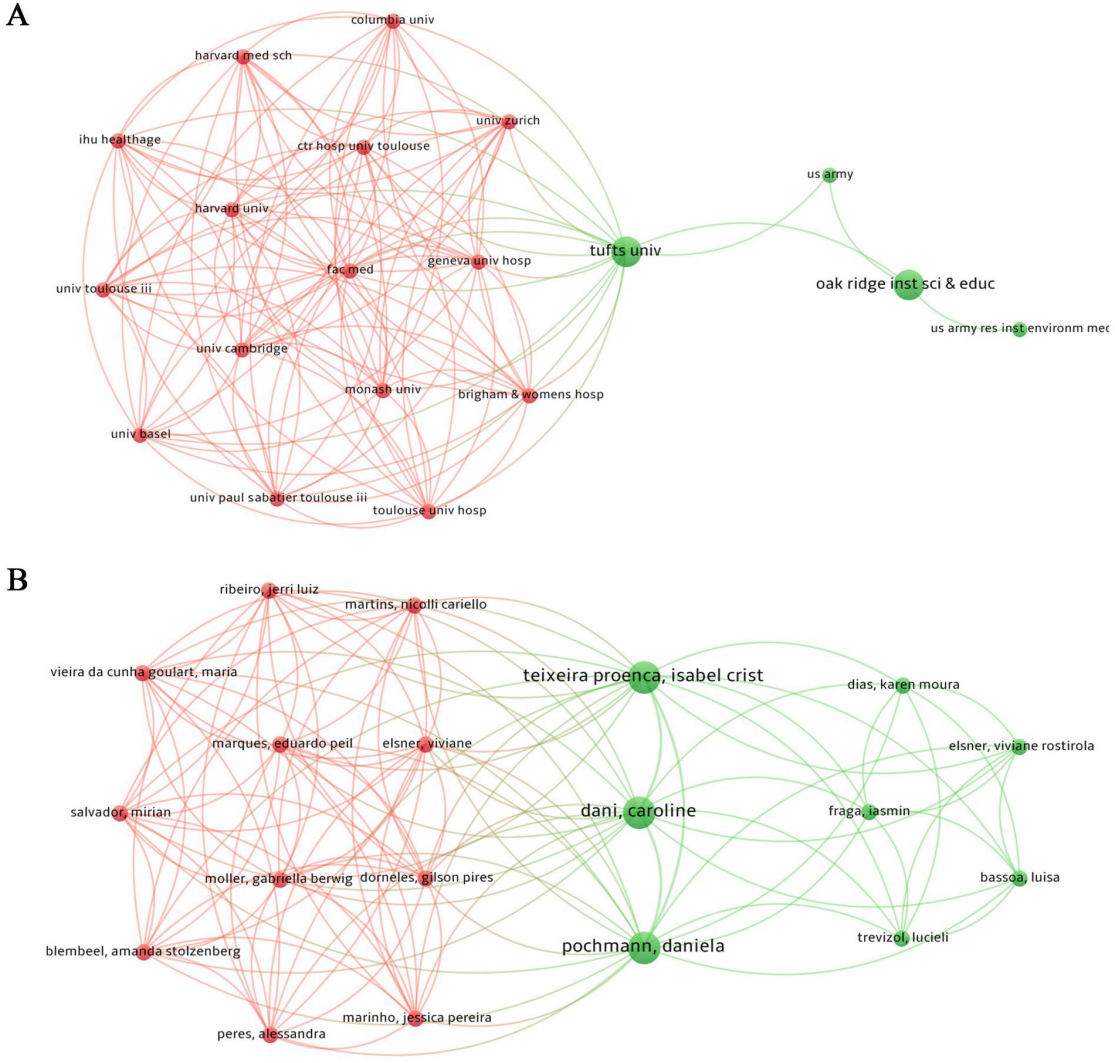
Core research institutions and author collaborations in combined interventions of exercise and nutrition. **(A)** Co-occurrence network of research institutions. **(B)** Co-occurrence network of core authors.

Publication data from 66 institutions across 16 countries were visualized using VOSviewer and Bibliometrix to describe collaboration patterns. In Figure 4A, node size represents national publication volume, Figure 4B illustrates relative research activity through color intensity, and Figure 4C depicts collaboration strength via link thickness. Countries such as the United States, the United Kingdom, Canada, and Australia are more frequently represented within collaborative linkages, although overall network density remains modest. Institutional analysis (Figure 5A) indicates that, among 66 institutions, 16 exhibit comparatively closer linkages. Nodes represent universities or research hospitals, with node size reflecting publication output and link thickness indicating collaboration intensity. The network appears concentrated within a limited subset of institutions, while cross-national integration remains relatively sparse. Author-level analysis (Figure 5B) shows that the 17 included studies involved 137 researchers. Nodes represent individual authors, with node size corresponding to publication output and colored links distinguishing collaborative subgroups. Three relatively distinct clusters are observable, centered around Dani Caroline, Pochmann Daniela, and Teixeira Proença Isabel Crist. Overall, the collaboration structure appears limited in scope, consistent with the exploratory and developing nature of this research area.

#### 3.3.3 Keyword co-occurrence analysis and clustering analysis

Betweenness centrality reflects a keyword’s capacity to connect different thematic areas within a knowledge network. Higher values indicate a greater bridging role across domains (49). Centrality analysis of 173 keywords identified six with non-zero values: Exercise, Inflammation, DNA methylation, Supplementation, Oxidative stress, and Skeletal muscle (Table 4). This pattern suggests that exercise may occupy an organizing position within the field, while inflammation and DNA methylation may act as connecting themes linking intervention strategies with biological discussion.

**TABLE 4:** High betweenness centrality keywords (B > 0) in the field of combined interventions of exercise and nutrition.

Rank	Node	Betweenness (B)	Frequency
1	Exercise	58.284	6
2	Inflammation	12.611	4
3	DNA methylation	12	3
4	Supplementation	6.13	3
5	Oxidative stress	2.159	3
6	Skeletal-muscle	1.816	3

**FIGURE 6:**
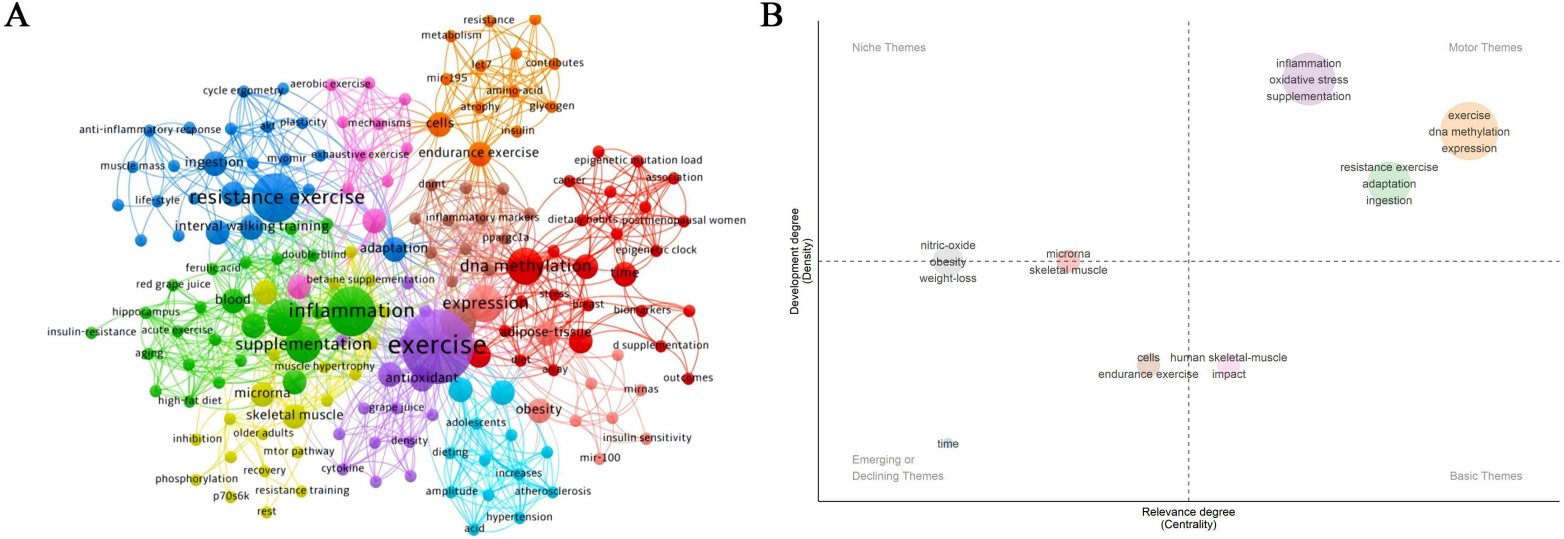
Keyword clustering analysis for the field of combined interventions of exercise and nutrition. **(A)** Keyword clustering map. **(B)** Keyword coordinate plot.

Keyword clustering analysis (Figure 6A) identified three overarching dimensions that correspond to the categories summarized in Table 5. (1) Intervention Strategies: This dimension reflects thematic groupings related to the structural configuration of combined exercise and nutrition protocols. Five clusters were identified: Exercise and Nutrition Integration (purple), Dietary Strategies within Combined Intervention (green), Endurance Oriented Intervention Design (orange), Training Modality Oriented Intervention Design (dark blue), and Aerobic Oriented Intervention Design (dark pink). The diversity observed within this dimension may primarily reflect heterogeneity in protocol design rather than uniform biological conclusions, underscoring the exploratory configuration of current interventions. (2) Mechanistic Exploration: This dimension captures themes related to biological processes discussed alongside combined interventions. Clusters include Epigenetic Regulation of Aging (red), Signal Transduction and Pathway Integration (yellow), and Epigenetic Regulation of Inflammation (brown). The co-occurrence of DNA methylation, epigenetic clock, phosphorylation, miRNAs, DNMT, and inflammatory markers suggests recurring areas of biological interest, rather than established mechanistic hierarchies or validated causal networks. (3) Health Outcomes: This dimension groups outcome-oriented themes reported in conjunction with epigenetic measures. Identified clusters include Cardiometabolic Health Outcomes (light blue) and Metabolic Health Related Outcomes (pale pink). The network proximity between outcome-related and mechanistic clusters may indicate that studies often interpret epigenetic observations in parallel with physiological indicators such as obesity, hypertension, and atherosclerosis. Nevertheless, these linkages remain exploratory, and consistent cross-population or cross-tissue patterns have not yet been clearly established.

**TABLE 5:** Keyword clustering themes in the field of combined interventions of exercise and nutrition.

Dimension	Color	Primary keyword	Research areas
Intervention strategies	Purple 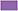	Exercise, antioxidant, grape juice	Exercise and nutrition integration
	Green 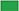	Inflammation, supplementation	Dietary strategies within combined intervention
	Orange 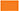	Endurance exercise, glycogen	Endurance oriented intervention design
	Dark blue 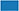	Resistance exercise, walking training	Training modality oriented intervention design
	Dark pink 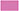	Aerobic exercise, exhaustive exercise	Aerobic oriented intervention design
Mechanistic exploration	Red 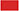	DNA methylation, epigenetic clock	Epigenetic regulation of aging
	Yellow 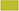	Phosphorylation, microrna	Signal transduction and pathway integration
	Brown 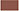	DNMT, inflammatory markers	Epigenetic regulation of inflammation
Health outcomes	Light blue 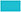	Dieting, hypertension, atherosclerosis	Cardiometabolic health outcomes
	Pale pink 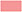	Expression, obesity, insulin sensitivity	Metabolic health related outcomes

The keyword strategy coordinate map (Figure 6B), based on “Relevance Degree” and “Development Degree,” provides additional thematic positioning (50). Motor themes in the first quadrant, such as “exercise,” “DNA methylation,” “ingestion,” and “inflammation,” appear to represent areas that are more frequently examined in epigenetic contexts. Basic themes in the second quadrant, including “human skeletal muscle” and “impact,” appear closely related to the field’s focus but comparatively less densely developed, which may indicate opportunities for further elaboration. Emerging or declining themes in the third quadrant, such as “cells” and “aging/time,” seem to reflect topics that are currently less integrated within broader thematic clusters. Niche themes in the fourth quadrant, including “nitric oxide” and “weight loss,” appear relatively developed within their own clusters but less centrally connected within the overall network. Taken together, the quadrant distribution may suggest that existing research tends to cluster around exercise- and nutrition-related epigenetic and inflammatory contexts, whereas integration across tissues, temporal dynamics, and cellular-level perspectives appears comparatively less prominent within the current keyword network. This pattern may highlight potential areas where future investigations could further strengthen biological integration and longitudinal depth.

## 4 Discussion

### 4.1 Overall overview and domain positioning

This review synthesizes available evidence on exercise and nutrition as epigenetic regulators and, through bibliometric analysis, outlines the current research landscape in this interdisciplinary field. The 17 included studies suggest that combined interventions of exercise and nutrition may be associated with a range of physiological outcomes, including metabolic health, muscular adaptation, inflammatory responses, and aging-related processes, within the populations and study contexts examined. These associations have primarily been discussed in relation to potential alterations in core epigenetic mechanisms, such as DNA methylation, histone modifications, and ncRNAs. Advances in epigenetic technologies may have facilitated exploratory investigations into molecular pathways underlying these interventions, contributing to an emerging conceptual framework for lifestyle-epigenome interactions rather than established translational applications. Overall, the evidence base remains largely exploratory and heterogeneous, and current findings should be interpreted as hypothesis-generating.

### 4.2 Interaction mechanisms in epigenetic multilayer regulatory networks

This review suggests that, based on current evidence across multiple studies, combined interventions of exercise and nutrition may be associated with more pronounced and potentially more stable epigenetic remodeling, although such interpretations remain exploratory. To illustrate this trend, we included the recent trial by Bischoff-Ferrari et al. (33), which compared three intervention arms: isolated resistance training, isolated omega-3 supplementation, and a combined intervention (33). The results indicated that the combined intervention was associated with more favorable changes in the GrimAge epigenetic clock relative to single-intervention arms within the specific study context. These findings suggest potential additive associations, rather than definitive superiority, when exercise and nutritional components are combined. This contrasts with earlier observational studies and small-scale trials within the DO-HEALTH framework, which primarily examined the independent effects of single interventions (e.g., vitamin D, omega-3, and exercise) on epigenetic aging markers or DNA methylation profiles (51–59). Together, these comparisons highlight emerging patterns, rather than conclusive evidence of synergy. Importantly, given differences in populations, intervention designs, and tissue or sample sources across studies, these patterns should not be interpreted as directly generalizable across populations or organs.

Similarly, in the context of glucose regulation, Aida et al. (38) reported that polyphenol-rich rice intake was associated with enhanced glycemic responses when combined with intermittent walking training, potentially mediated through changes in NFKB2 methylation (38). Notably, this association was less evident in monotherapy groups, suggesting a potentially additive pattern within the study context that warrants further investigation. Taken together, these observations suggest that combined interventions of exercise and nutrition may offer potential advantages over single interventions within certain contexts, although the overall evidence remains preliminary and hypothesis-generating.

From a longitudinal perspective, earlier studies primarily examined associations between post-intervention miRNA changes and phenotypic outcomes (39–42, 45), largely within short-term or single-time-point designs. More recent studies by Heianza et al. (35) and Margolis et al. (47) reported that miR-99/100 and members of the let-7 family were associated with fat loss and muscle-related adaptations following combined interventions (35, 44), and may represent responsive epigenetic signals relevant for hypothesis generation. Importantly, these miRNAs should not be interpreted as validated predictive biomarkers. Rather, within the current exploratory evidence base, they serve as preliminary signals that may help generate hypotheses regarding molecular processes potentially contributing to inter-individual variability in intervention responses, primarily within the specific tissues or biospecimens assessed (e.g., blood-derived samples or skeletal muscle). Overall, these findings suggest that the field may reflect increasing attention to temporally informed lines of inquiry beyond purely descriptive observations.

At the same time, interpretation of these emerging patterns requires careful consideration of variability and uncertainty within the current evidence base. Across the included studies, epigenetic responses to combined interventions were not consistently observed, and several investigations reported null or context-dependent findings. Such variability may reflect both biological specificity and methodological factors, including differences in intervention duration, sampling timing, tissue or biospecimen source, and analytical platform sensitivity. In some reports, circulating miRNAs and acute exercise-related signals appear to behave as relatively rapid and potentially transient markers, while gene-specific DNA methylation changes and DNA methylation-based aging indicators have been discussed as showing more temporally persistent patterns in certain contexts (40). However, direct evidence supporting long-term stability remains limited. Notably, evidence regarding histone modifications is especially sparse and inconsistent, with mixed or null findings reported across the small number of available studies, suggesting that observed histone acetylation patterns may depend on tissue type, intervention protocol, and population characteristics (37, 46). Collectively, these considerations suggest that current observations are best interpreted as exploratory and hypothesis-generating, rather than as stable, causal, or broadly generalizable epigenetic effects across populations or tissues.

### 4.3 Global collaborative patterns, research hotspots and frontier trends based on bibliometric analysis

Research examining combined interventions of exercise and nutrition in relation to health outcomes within epigenetic contexts appears to be gradually extending beyond initial descriptive observations in some studies, with increasing attention to mechanistic themes. However, the overall evidence base remains limited in size and heterogeneous in scope. Although the cumulative number of publications has increased over time, the modest volume of eligible empirical studies suggests that this field remains at an exploratory stage.

Across the 17 included studies comprising 1,568 participants, several recurring methodological characteristics can be noted. First, randomized controlled trials represent the predominant design, accounting for 13 studies (76.5%) (32–44). While this distribution may indicate an effort to strengthen internal validity, many trials remain small in scale and focused on short-term outcomes. Second, study size appears polarized: 11 investigations are small-sample exploratory studies (34, 37, 39–47), whereas only one study represents a larger-scale confirmatory design (33). This pattern may suggest that the field is still largely oriented toward hypothesis generation rather than definitive clinical translation. Third, although multiple age groups are represented, signals of responsiveness have more frequently been reported in populations such as older adults or athletic cohorts. These observations should be interpreted cautiously, as baseline health status, training adaptation, and sampling strategies may influence detectability of epigenetic changes (33, 34, 37, 38, 40, 42, 43, 45, 46). Broader inclusion of populations with diverse health conditions, lifestyles, and genetic backgrounds may help clarify whether observed patterns extend beyond currently studied groups (33, 34, 37, 38, 40, 42, 43, 45, 46).

From a global perspective, publication output and collaboration networks appear concentrated in high-income countries, particularly the United States. This distribution may reflect disparities in research infrastructure and availability of interdisciplinary research platforms. Concentration of expertise in standardized intervention protocols and laboratory methodologies may also contribute to geographic clustering (60, 61). At the same time, limited representation of low- and middle-income populations may restrict the contextual breadth of the current evidence base. Environmental exposures, sociocultural factors, and healthcare access vary substantially across regions and may influence epigenetic responsiveness (62–64). Expanding collaborative networks and supporting inclusive research infrastructures may therefore enhance representativeness and external validity, while reducing potential knowledge gaps related to underrepresented populations.

Bibliometric approaches, including keyword clustering, strategic coordinate mapping, and overlay visualization, provide a descriptive mapping of how themes co-occur within the current literature. Rather than representing a consolidated conceptual framework, these patterns may reflect how investigators have provisionally organized inquiry around combined interventions of exercise and nutrition in relation to epigenetic contexts and associated health outcomes. Three thematic dimensions can be described based on keyword clustering. The first dimension, intervention strategy configuration, highlights heterogeneity in protocol design. Studies frequently combine aerobic or resistance exercise with specific nutritional components, such as omega-3 fatty acids or polyphenols. This diversity may indicate that methodological experimentation remains prominent and that no standardized or convergent intervention model has yet emerged. The second dimension, mechanistic exploration, groups themes related to DNA methylation, miRNAs regulation, and inflammation-associated signaling pathways. The recurrent co-occurrence of these keywords may suggest areas of thematic interest where biological processes are discussed alongside intervention strategies. However, such clustering should be interpreted as descriptive proximity rather than as evidence of clearly defined mechanistic hierarchies, coordinated multi-layer regulation, or reproducible causal pathways. The third dimension, health-related endpoints, includes aging-related indicators, metabolic parameters, and body composition measures. Their proximity to intervention and mechanistic clusters in the network may reflect a tendency for studies to interpret epigenetic observations in parallel with physiological outcomes. Nevertheless, these linkages remain exploratory, and consistent cross-population or cross-tissue patterns have not yet been clearly established.

Recent increases in attention to terms such as miRNAs, obesity, and weight loss may indicate expanding interest in metabolically relevant contexts, particularly given the global burden of obesity-related disorders (65, 66). At present, however, the extent to which intervention-associated epigenetic changes reproducibly relate to obesity phenotypes remains only partially characterized within the current literature (65, 66). These areas may therefore represent potential directions for future validation rather than confirmed translational domains.

Within the strategic coordinate map, themes such as epigenetic clock, mTOR signaling pathway, and tissue-specific responses appear positioned in quadrants that may suggest developmental potential. This positioning could reflect emerging attention to temporality, pathway integration, and tissue specificity. At the same time, it highlights several unresolved questions. The durability of epigenetic changes over time and the quantification of inter-individual variability remain incompletely understood. Addressing these uncertainties may require larger and longer-term studies, harmonized analytic pipelines, and replication across diverse tissues and populations. Methodological advances, including single-cell epigenomics, spatial multi-omics integration, and computational modeling, may offer additional tools for hypothesis generation. However, their application in this field remains at an early stage, and any predictive or precision-oriented implications would require cautious interpretation pending further validation and reproducibility.

### 4.4 Future directions and challenges

To strengthen evidence mapping and inform longer-term translation, research on combined interventions of exercise and nutrition in relation to epigenetic contexts and associated health outcomes may benefit from progress in four areas.

(1) Advancing standardization and methodological development in epigenetic assessment may improve cross-study comparability. Current research shows substantial heterogeneity in detection methods, target selection, and analytical workflows (32, 33, 41, 45). Notably, the two available studies assessing histone modifications reported inconsistent findings (37, 46), underscoring the limited and heterogeneous evidence base in this domain and suggesting that reported patterns may vary by tissue type, intervention characteristics, and baseline participant conditions. A traceable and standardized approach to epigenetic measurement could therefore be considered, including harmonized procedures for sample processing, assay parameters, and analytic pipelines. Single-cell epigenomics and spatiotemporal multi-omics approaches may further help characterize tissue- and cell-type-specific patterns. Computational modeling, including machine learning, may assist in identifying candidate features within complex datasets, provided that models are transparently reported and validated across independent cohorts and biospecimen types.

(2) Broadening tissue coverage and clarifying cross-tissue relevance may help determine whether observed patterns extend beyond commonly sampled biospecimens. Most existing studies rely on blood as an accessible source of epigenetic measures (32, 35, 45) or focus on skeletal muscle, which is directly engaged by exercise, to characterize local responses (39, 40). While informative within the sampled context, epigenetic patterns may vary across tissues and cell types, and findings from blood or skeletal muscle may not fully reflect processes in other metabolically relevant organs. For example, hepatocyte-related patterns may relate to glucose and lipid homeostasis, whereas adipose-related patterns could relate to adipokine signaling and inflammatory regulation (67, 68). Epigenetic dynamics in brain regions involved in metabolic or stress-related pathways may also contribute indirectly to systemic outcomes (69). Conceptually, inter-organ coordination, if present, could involve circulating mediators such as extracellular vesicles, cytokines, or metabolic intermediates (70); however, current evidence remains insufficient to support consistent cross-tissue pathways. Multi-tissue sampling strategies, where feasible, may help clarify the scope and limits of circulating readouts.

(3) Broadening outcome assessment frameworks may help interpret epigenetic findings within wider health and societal contexts. Although existing studies have reported epigenetic measures alongside multiple physiological domains, reliance on single-indicator systems may provide only a partial view of broader impacts. Dimensions such as socioeconomic burden, psychological resilience, and long-term quality of life remain comparatively underexplored. It may be useful for future research to consider more integrative assessment frameworks that examine physiological measures and epigenetic readouts alongside validated psychological indicators, measures of social functioning, and selected health-economic outcomes, where feasible. For obesity-related combined interventions, for instance, incorporating quality-of-life instruments (e.g., SF-36) and contextual cost analyses may offer additional interpretive depth (71, 72).

(4) Incorporating longitudinal designs and heterogeneity-aware analyses may strengthen understanding of durability and context dependence. Many included studies used short-term or single-time-point designs, which limits inference about persistence and temporal dynamics (33, 34, 36, 40, 41). Multi-time-point longitudinal studies may help examine time-dependent patterns of epigenetic responsiveness. In parallel, variables such as ethnicity, sex, age, baseline health status, and living environment may help clarify context-specific variability. For example, Bischoff-Ferrari et al. (33) reported that observed patterns related to vitamin D and omega-3 supplementation varied with baseline nutritional status and sunlight exposure (33). Fiore et al. (73) further discussed that response variability in metabolic syndrome or pediatric obesity contexts may relate to baseline nutritional status, gut microbiota composition, and epigenetic sensitivity (73). Together, these observations highlight the potential value of longitudinal and heterogeneity-aware designs, while reinforcing the need to avoid overgeneralization across populations or tissues.

Implementation and evidence constraints should also be acknowledged when discussing longer-term translational possibilities. Sustained combined interventions may face adherence challenges and real-world variability, which can complicate interpretation of longer-term trajectories and limit comparability across studies. In addition, epigenetic profiling and multi-omics workflows remain resource-intensive, with access influenced by laboratory infrastructure, technical expertise, and associated costs (74). These considerations may be particularly relevant in low and middle-income countries, where scalability and sustained follow-up capacity can be constrained (75, 76). Cross-platform variability and uncertain clinical interpretability may further limit near-term application (74, 77). Ethical considerations are also pertinent, as epigenetic data may raise issues related to privacy, governance, and secondary use, and models derived from unrepresentative cohorts may risk amplifying bias or widening disparities if implemented without safeguards (74, 78). In this context, translational discussions may warrant transparent reporting, context-aware interpretation, validation across diverse populations and biospecimen types, privacy-conscious data practices, and explicit attention to equity and accessibility.

Within this broader translational landscape, a cautious applied perspective may also be relevant for sport nutrition. In athletic or physically trained cohorts, available studies have reported short-term, context-dependent shifts in ncRNA-related readouts and selected DNA methylation targets in blood-derived samples or skeletal muscle, although findings remain heterogeneous and sensitive to sampling windows and assay characteristics (34, 39–42, 44–47). These observations may serve as preliminary reference points for future athlete-focused trials that prespecify tissues, harmonize assays, and test clearly defined questions such as nutrient timing, recovery-phase supplementation, and training-load interactions. Longer-duration studies using DNA methylation-based aging indicators have also been discussed as potential monitoring tools within clearly delimited contexts (33, 36). Importantly, current findings are not practice-ready and should not be interpreted as prescriptive guidance or broadly generalizable across populations or tissues.

## 5 Limitations

Several limitations should be considered when interpreting the findings of this review. First, the overall evidence base remains limited, as only a relatively small number of eligible studies were identified following searches across multiple databases and application of predefined inclusion criteria. This constrains the breadth of evidence mapped and limits the strength of overarching trends. Consistent with the exploratory nature of a scoping review, no quantitative synthesis or formal methodological quality appraisal was conducted, which may limit inferential interpretation. Second, substantial heterogeneity was observed across study populations, including healthy individuals, athletes, and participants with metabolic conditions. Most studies relied primarily on blood and/or skeletal muscle-derived samples, which are commonly accessible in human research. While these tissues are practical for sampling, they may not fully capture epigenetic regulation occurring in other metabolically relevant tissues, such as the liver, adipose tissue, or brain. Accordingly, extrapolation beyond the sampled tissues should be undertaken cautiously. Third, methodological variability across studies represents an additional limitation. Differences in analytical platforms and assays used to assess DNA methylation, histone modifications, and non-coding RNAs may introduce measurement-related heterogeneity, thereby limiting direct comparability across studies. Furthermore, most interventions were short-term in duration, ranging from acute exposures to several months, which restricts insight into the long-term stability or persistence of reported epigenetic changes. Finally, findings from the scientometric analysis should be interpreted with caution. The relatively small number of included studies may influence clustering stability and thematic mapping. In addition, results may be affected by database selection, language restrictions, exclusion of qualitative literature, and potential sampling bias.

## 6 Conclusion

This scoping review synthesizes current evidence on combined interventions of exercise and nutrition in relation to epigenetic responsiveness and health-related outcomes. Available studies suggest that such interventions may be associated with concurrent changes in epigenetic markers, including DNA methylation, histone modifications, and ncRNAs, alongside alterations in metabolic, muscular, inflammatory, and aging-related parameters. However, the existing literature remains largely exploratory, with common limitations including small sample sizes, heterogeneous intervention designs, and limited longitudinal follow-up. Consequently, the stability, generalizability, and functional relevance of these epigenetic responses have yet to be clearly established, particularly across diverse populations and broader health contexts. In addition, most evidence is derived from blood-based samples and/or skeletal muscle, and extrapolation to other tissues or organs should therefore be made with caution. Future research may benefit from prioritizing standardized epigenetic assessments, longitudinal and multi-tissue study designs, and integrative analytical approaches to further explore underlying biological processes and contextualize epigenetic observations within specific study settings.

## Data Availability

The original contributions presented in this study are included in this article/Supplementary material, further inquiries can be directed to the corresponding author.
