# Supplementary material for: Exercise and nutrition as epigenetic regulators of gene expression: an exploratory scoping review with bibliometric analysis
Source: Front Nutr. 2026 Mar 10;13:1773920. doi: 10.3389/fnut.2026.1773920 (PMC13008672; doi:10.3389/fnut.2026.1773920)
Supplement: Supplementary file 2 [file Data_Sheet_2.pdf]

1.1 PubMed

Search	Query
#1	(("Exercise"[Mesh] OR "Motor Activity"[Mesh] OR "Physical Exertion "[Mesh] OR "Sports"[Mesh] OR exercise[Title/Abstract] OR "physical activity"[Title/Abstract] OR training[Title/Abstract] OR workout[Title/Abstract]))
#2	(("Nutrition Therapy"[Mesh] OR "Diet Therapy"[Mesh] OR "Nutritional Support"[Mesh] OR "Dietary Supplements"[Mesh] OR "Food, Formulated"[Mesh] OR "Health Promotion"[Mesh] OR "Counseling"[Mesh] OR "Nutrition intervention"[Title/Abstract] OR "Dietary intervention"[Title/Abstract] OR "Nutritional care"[Title/Abstract] OR "Nutritional support"[Title/Abstract] OR "Diet therapy"[Title/Abstract] OR "Nutrition therapy"[Title/Abstract] OR "Dietary modification"[Title/Abstract] OR "Food-based intervention"[Title/Abstract] OR Supplementation[Title/Abstract] OR "Medical nutrition therapy"[Title/Abstract] OR "Nutritional counseling"[Title/Abstract] OR "Dietary management"[Title/Abstract] OR "Dietary advice"[Title/Abstract] OR "Nutrition education"[Title/Abstract]))
#3	(("DNA Methylation"[Mesh] OR "CpG Islands"[Mesh] OR "Epigenesis, Genetic"[Mesh] OR "DNA methylation"[Title/Abstract] OR "DNA methylated"[Title/Abstract] OR "DNA methylome"[Title/Abstract] OR "methylation pattern"[Title/Abstract] OR "methylation profile"[Title/Abstract] OR "methylation status"[Title/Abstract] OR "CpG methylation"[Title/Abstract] OR "CpG site"[Title/Abstract] OR "CpG island"[Title/Abstract] OR "5-methylcytosine"[Title/Abstract] OR "5mC"[Title/Abstract] OR "epigenetic modification"[Title/Abstract] OR "epigenetic marker"[Title/Abstract] OR methyltransferase[Title/Abstract] OR DNMT[Title/Abstract] OR "DNA methyltransferase"[Title/Abstract] OR methylome[Title/Abstract] OR "methylation analysis"[Title/Abstract] OR "methylation sequencing"[Title/Abstract] OR "methylation array"[Title/Abstract] OR "methylation level"[Title/Abstract] OR hypermethylation[Title/Abstract] OR hypomethylation[Title/Abstract] OR "global methylation"[Title/Abstract] OR "genome-wide methylation"[Title/Abstract] OR "methylation biomarker"[Title/Abstract]))

**#4**	(("Histone Code"[Mesh] OR "Histones"[Mesh] OR "Protein Processing, Post-Translational"[Mesh] OR "Epigenesis, Genetic"[Mesh] OR "Chromatin Assembly and Disassembly"[Mesh] OR "Histone modification"[Title/Abstract] OR "Histone methylation"[Title/Abstract] OR "Histone acetylation"[Title/Abstract] OR "Histone phosphorylation"[Title/Abstract] OR "Histone ubiquitination"[Title/Abstract] OR "Histone mark"[Title/Abstract] OR "Histone code"[Title/Abstract] OR "Histone tail"[Title/Abstract] OR "Histone H3"[Title/Abstract] OR "Histone H4"[Title/Abstract] OR H3K4me3[Title/Abstract] OR H3K27ac[Title/Abstract] OR H3K9me3[Title/Abstract] OR H3K36me3[Title/Abstract] OR "Histone methyltransferase"[Title/Abstract] OR "Histone acetyltransferase"[Title/Abstract] OR "Histone deacetylase"[Title/Abstract] OR HDAC[Title/Abstract] OR HAT[Title/Abstract] OR HMT[Title/Abstract] OR "Chromatin modification"[Title/Abstract] OR "Chromatin remodeling"[Title/Abstract] OR "Epigenetic modification"[Title/Abstract] OR "Lysine methylation"[Title/Abstract] OR "Lysine acetylation"[Title/Abstract] OR "Histone variant"[Title/Abstract]))
**#5**	(("RNA, Untranslated"[Mesh] OR "MicroRNAs"[Mesh] OR "RNA, Long Noncoding"[Mesh] OR "RNA, Circular"[Mesh] OR "RNA, Small Interfering"[Mesh] OR "RNA, Ribosomal"[Mesh] OR "RNA, Transfer"[Mesh] OR "RNA, Small Nuclear"[Mesh] OR "RNA, Antisense"[Mesh] OR "Epigenesis, Genetic"[Mesh] OR "Non-coding RNA"[Title/Abstract] OR "Noncoding RNA"[Title/Abstract] OR ncRNA[Title/Abstract] OR "MicroRNA"[Title/Abstract] OR miRNA[Title/Abstract] OR "Long non-coding RNA"[Title/Abstract] OR "Long noncoding RNA"[Title/Abstract] OR lncRNA[Title/Abstract] OR "Circular RNA"[Title/Abstract] OR circRNA[Title/Abstract] OR "Small interfering RNA"[Title/Abstract] OR siRNA[Title/Abstract] OR "Ribosomal RNA"[Title/Abstract] OR rRNA[Title/Abstract] OR "Transfer RNA"[Title/Abstract] OR tRNA[Title/Abstract] OR "Small nuclear RNA"[Title/Abstract] OR snRNA[Title/Abstract] OR "Small nucleolar RNA"[Title/Abstract] OR snoRNA[Title/Abstract] OR "Piwi-interacting RNA"[Title/Abstract] OR piRNA[Title/Abstract] OR "Antisense RNA"[Title/Abstract] OR "Enhancer RNA"[Title/Abstract] OR "Regulatory RNA"[Title/Abstract]))

**#6**	(("Treatment Outcome"[Mesh] OR "Patient Outcome Assessment"[Mesh] OR "Morbidity"[Mesh] OR "Mortality"[Mesh] OR "Survival Rate"[Mesh] OR "Quality of Life"[Mesh] OR "Physical Fitness"[Mesh] OR "Exercise Tolerance"[Mesh] OR "Recovery of Function"[Mesh] OR " Health Status Indicators"[Mesh] OR "Efficiency"[Mesh] OR Efficacy[Title/Abstract] OR Effectiveness[Title/Abstract] OR Outcome[Title/Abstract] OR Outcomes[Title/Abstract] OR "Health outcome"[Title/Abstract] OR "Performance outcome"[Title/Abstract] OR "Clinical outcome"[Title/Abstract] OR "Functional outcome"[Title/Abstract] OR Morbidity[Title/Abstract] OR Mortality[Title/Abstract] OR Survival[Title/Abstract] OR "Quality of life"[Title/Abstract] OR QoL[Title/Abstract] OR "Physical performance"[Title/Abstract] OR "Exercise performance"[Title/Abstract] OR "Athletic performance"[Title/Abstract] OR "Work performance"[Title/Abstract] OR "Cognitive performance"[Title/Abstract] OR Recovery[Title/Abstract] OR "Functional recovery"[Title/Abstract] OR "Symptom severity"[Title/Abstract] OR Hospitalization[Title/Abstract] OR Rehospitalization[Title/Abstract] OR Biomarker[Title/Abstract] OR Endpoint[Title/Abstract] OR "Physical function"[Title/Abstract] OR "Cognitive function"[Title/Abstract] OR "Peak VO2"[Title/Abstract] OR VO2max[Title/Abstract] OR "One-repetition maximum"[Title/Abstract] OR 1RM[Title/Abstract]))
**#7**	**#7：(#1 AND #2) AND (#3 OR #4 OR #5) AND #6**<br>((("Exercise"[Mesh] OR "Motor Activity"[Mesh] OR "Physical Exertion"[Mesh] OR "Sports"[Mesh] OR exercise[Title/Abstract] OR "physical activity"[Title/Abstract] OR training[Title/Abstract] OR workout[Title/Abstract]) **AND** ("Nutrition Therapy"[Mesh] OR "Diet Therapy"[Mesh] OR "Nutritional Support"[Mesh] OR "Dietary Supplements"[Mesh] OR "Food, Formulated"[Mesh] OR "Health Promotion"[Mesh] OR "Counseling"[Mesh] OR "Nutrition intervention"[Title/Abstract] OR "Dietary intervention"[Title/Abstract] OR "Nutritional care"[Title/Abstract] OR "Nutritional support"[Title/Abstract] OR "Diet therapy"[Title/Abstract] OR "Nutrition therapy"[Title/Abstract] OR "Dietary modification"[Title/Abstract] OR "Food-based intervention"[Title/Abstract] OR Supplementation[Title/Abstract] OR "Medical nutrition therapy"[Title/Abstract] OR "Nutritional counseling"[Title/Abstract] OR "Dietary management"[Title/Abstract] OR "Dietary advice"[Title/Abstract] OR "Nutrition education"[Title/Abstract])) **AND** ((("DNA Methylation"[Mesh] OR "CpG Islands"[Mesh] OR "Epigenesis, Genetic"[Mesh] OR "DNA methylation"[Title/Abstract] OR "DNA methylated"[Title/Abstract] OR "DNA methylome"[Title/Abstract] OR "methylation pattern"[Title/Abstract] OR "methylation profile"[Title/Abstract] OR "methylation status"[Title/Abstract] OR "CpG methylation"[Title/Abstract] OR "CpG site"[Title/Abstract] OR "CpG island"[Title/Abstract] OR "5-methylcytosine"[Title/Abstract] OR "5mC"[Title/Abstract] OR "epigenetic modification"[Title/Abstract] OR

	"epigenetic marker"[Title/Abstract] OR methyltransferase[Title/Abstract] OR DNMT[Title/Abstract] OR "DNA methyltransferase"[Title/Abstract] OR methylome[Title/Abstract] OR "methylation analysis"[Title/Abstract] OR "methylation sequencing"[Title/Abstract] OR "methylation array"[Title/Abstract] OR "methylation level"[Title/Abstract] OR hypermethylation[Title/Abstract] OR hypomethylation[Title/Abstract] OR "global methylation"[Title/Abstract] OR "genome-wide methylation"[Title/Abstract] OR "methylation biomarker"[Title/Abstract]) **OR** ("Histone Code"[Mesh] OR "Histones"[Mesh] OR "Protein Processing, Post-Translational"[Mesh] OR "Epigenesis, Genetic"[Mesh] OR "Chromatin Assembly and Disassembly"[Mesh] OR "Histone modification"[Title/Abstract] OR "Histone methylation"[Title/Abstract] OR "Histone acetylation"[Title/Abstract] OR "Histone phosphorylation"[Title/Abstract] OR "Histone ubiquitination"[Title/Abstract] OR "Histone mark"[Title/Abstract] OR "Histone code"[Title/Abstract] OR "Histone tail"[Title/Abstract] OR "Histone H3"[Title/Abstract] OR "Histone H4"[Title/Abstract] OR H3K4me3[Title/Abstract] OR H3K27ac[Title/Abstract] OR H3K9me3[Title/Abstract] OR H3K36me3[Title/Abstract] OR "Histone methyltransferase"[Title/Abstract] OR "Histone acetyltransferase"[Title/Abstract] OR "Histone deacetylase"[Title/Abstract] OR HDAC[Title/Abstract] OR HAT[Title/Abstract] OR HMT[Title/Abstract] OR "Chromatin modification"[Title/Abstract] OR "Chromatin remodeling"[Title/Abstract] OR "Epigenetic modification"[Title/Abstract] OR "Lysine methylation"[Title/Abstract] OR "Lysine acetylation"[Title/Abstract] OR "Histone variant"[Title/Abstract]) **OR** ("RNA, Untranslated"[Mesh] OR "MicroRNAs"[Mesh] OR "RNA, Long Noncoding"[Mesh] OR "RNA, Circular"[Mesh] OR "RNA, Small Interfering"[Mesh] OR "RNA, Ribosomal"[Mesh] OR "RNA, Transfer"[Mesh] OR "RNA, Small Nuclear"[Mesh] OR "RNA, Antisense"[Mesh] OR "Epigenesis, Genetic"[Mesh] OR "Non-coding RNA"[Title/Abstract] OR "Noncoding RNA"[Title/Abstract] OR ncRNA[Title/Abstract] OR "MicroRNA"[Title/Abstract] OR miRNA[Title/Abstract] OR "Long non-coding RNA"[Title/Abstract] OR "Long noncoding RNA"[Title/Abstract] OR lncRNA[Title/Abstract] OR "Circular RNA"[Title/Abstract] OR circRNA[Title/Abstract] OR "Small interfering RNA"[Title/Abstract] OR siRNA[Title/Abstract] OR "Ribosomal RNA"[Title/Abstract] OR rRNA[Title/Abstract] OR "Transfer RNA"[Title/Abstract] OR tRNA[Title/Abstract] OR "Small nuclear RNA"[Title/Abstract] OR snRNA[Title/Abstract] OR "Small nucleolar RNA"[Title/Abstract] OR snoRNA[Title/Abstract] OR "Piwi-interacting RNA"[Title/Abstract] OR piRNA[Title/Abstract] OR "Antisense RNA"[Title/Abstract] OR "Enhancer RNA"[Title/Abstract] OR "Regulatory RNA"[Title/Abstract]))) **AND** ("Treatment Outcome"[Mesh] OR "Patient Outcome Assessment"[Mesh] OR "Morbidity"[Mesh] OR "Mortality"[Mesh] OR "Survival Rate"[Mesh] OR "Quality of Life"[Mesh] OR "Physical Fitness"[Mesh] OR "Exercise T

	olerance"[Mesh] OR "Recovery of Function"[Mesh] OR "Health Status Indicators"[Mesh] OR "Efficiency"[Mesh] OR Efficacy[Title/Abstract] OR Effectiveness[Title/Abstract] OR Outcome[Title/Abstract] OR Outcomes[Title/Abstract] OR "Health outcome"[Title/Abstract] OR "Performance outcome"[Title/Abstract] OR "Clinical outcome"[Title/Abstract] OR "Functional outcome"[Title/Abstract] OR Morbidity[Title/Abstract] OR Mortality[Title/Abstract] OR Survival[Title/Abstract] OR "Quality of life"[Title/Abstract] OR QoL[Title/Abstract] OR "Physical performance"[Title/Abstract] OR "Exercise performance"[Title/Abstract] OR "Athletic performance"[Title/Abstract] OR "Work performance"[Title/Abstract] OR "Cognitive performance"[Title/Abstract] OR Recovery[Title/Abstract] OR "Functional recovery"[Title/Abstract] OR "Symptom severity"[Title/Abstract] OR Hospitalization[Title/Abstract] OR Rehospitalization[Title/Abstract] OR Biomarker[Title/Abstract] OR Endpoint[Title/Abstract] OR "Physical function"[Title/Abstract] OR "Cognitive function"[Title/Abstract] OR "Peak VO2"[Title/Abstract] OR VO2max[Title/Abstract] OR "One-repetition maximum"[Title/Abstract] OR 1RM[Title/Abstract]))
**Filters:**	Adaptive Clinical Trial, Classical Article, Clinical Study, Clinical Trial, Comparative Study, Controlled Clinical Trial, Multicenter Study, Observational Study, Pragmatic Clinical Trial, Randomized Controlled Trial, English

1.2 Web of Science

Search	Query
**#1**	TS=(("Exercise" OR "Motor Activity" OR "Physical Exertion" OR "S ports" OR exercise OR "physical activity" OR training OR workout))
**#2**	TS=(("Nutrition Therapy" OR "Diet Therapy" OR "Nutritional Suppor t" OR "Dietary Supplements" OR "Food Formulated" OR "Health Pr omotion" OR "Counseling" OR "Nutrition intervention*" OR "Dietary intervention*" OR "Nutritional care" OR "Nutritional support" OR " Diet therapy" OR "Nutrition therapy" OR "Dietary modification*" OR "Food-based intervention*" OR Supplementation OR "Medical nutriti on therapy" OR "Nutritional counseling" OR "Dietary management" OR "Dietary advice" OR "Nutrition education"))
**#3**	TS=(("DNA Methylation" OR "CpG Islands" OR "Epigenesis Genetic " OR "DNA methylation" OR "DNA methylated" OR "DNA methylo me" OR "methylation pattern" OR "methylation profile" OR "methyla tion status" OR "CpG methylation" OR "CpG site" OR "CpG island" OR "5-methylcytosine" OR "5mC" OR "epigenetic modification" OR "epigenetic marker" OR methyltransferase OR DNMT OR "DNA me thyltransferase" OR methylome OR "methylation analysis" OR "methy lation sequencing" OR "methylation array" OR "methylation level" O R hypermethylation OR hypomethylation OR "global methylation" O R "genome-wide methylation" OR "methylation biomarker"))
**#4**	TS=(("Histone Code" OR "Histones" OR "Protein Processing Post-Tra nslational" OR "Epigenesis Genetic" OR "Chromatin Assembly and D isassembly" OR "Histone modification*" OR "Histone methylation" O R "Histone acetylation" OR "Histone phosphorylation" OR "Histone u biquitination" OR "Histone mark*" OR "Histone code" OR "Histone tail" OR "Histone H3" OR "Histone H4" OR H3K4me3 OR H3K27a c OR H3K9me3 OR H3K36me3 OR "Histone methyltransferase" OR "Histone acetyltransferase" OR "Histone deacetylase" OR HDAC OR HAT OR HMT OR "Chromatin modification" OR "Chromatin remo deling" OR "Epigenetic modification" OR "Lysine methylation" OR " Lysine acetylation" OR "Histone variant"))
**#5**	TS=(("RNA Untranslated" OR "MicroRNAs" OR "RNA Long Noncod ing" OR "RNA Circular" OR "RNA Small Interfering" OR "RNA Ri bosomal" OR "RNA Transfer" OR "RNA Small Nuclear" OR "RNA Antisense" OR "Epigenesis Genetic" OR "Non-coding RNA" OR "No ncoding RNA" OR ncRNA OR "MicroRNA" OR miRNA OR "Long non-coding RNA" OR "Long noncoding RNA" OR lncRNA OR "Ci rcular RNA" OR circRNA OR "Small interfering RNA" OR siRNA

	OR "Ribosomal RNA" OR rRNA OR "Transfer RNA" OR tRNA OR "Small nuclear RNA" OR snRNA OR "Small nucleolar RNA" OR snoRNA OR "Piwi-interacting RNA" OR piRNA OR "Antisense RNA" OR "Enhancer RNA" OR "Regulatory RNA"))
**#6**	TS=(("Treatment Outcome" OR "Patient Outcome Assessment" OR "Morbidity" OR "Mortality" OR "Survival Rate" OR "Quality of Life" OR "Physical Fitness" OR "Exercise Tolerance" OR "Recovery of Function" OR "Health Status Indicators" OR "Efficiency" OR Efficacy OR Effectiveness OR Outcome OR Outcomes OR "Health outcome*" OR "Performance outcome*" OR "Clinical outcome*" OR "Functional outcome*" OR Morbidity OR Mortality OR Survival OR "Quality of life" OR QoL OR "Physical performance" OR "Exercise performance" OR "Athletic performance" OR "Work performance" OR "Cognitive performance" OR Recovery OR "Functional recovery" OR "Symptom severity" OR Hospitalization OR Rehospitalization OR Biomarker* OR Endpoint OR Endpoints OR "Physical function" OR "Cognitive function" OR "Peak VO2" OR VO2max OR "One-repetition maximum" OR 1RM))
**#7**	**#7：(#1 AND #2) AND (#3 OR #4 OR #5) AND #6**<br>TS=((exercise OR "physical activity" OR training OR workout) AND ("nutrition therapy" OR "diet therapy" OR "nutritional support" OR "dietary supplement*" OR "food formulated" OR "health promotion" OR counseling OR "nutrition intervent*" OR "dietary intervent*" OR "nutritional care" OR "diet therapy" OR "nutrition therapy" OR "dietary modific*" OR "food-based intervent*" OR supplementation OR "medical nutrition therapy" OR "nutritional counseling" OR "dietary management" OR "dietary advice" OR "nutrition education") AND (("DNA methylation" OR "CpG island*" OR "epigenesis genetic" OR "DNA methylated" OR "DNA methylome" OR "methylation pattern" OR "methylation profile" OR "methylation status" OR "CpG methylation" OR "CpG site" OR "5-methylcytosine" OR "5mC" OR "epigenetic modification" OR "epigenetic marker" OR methyltransferase OR DNMT OR "DNA methyltransferase" OR methylome OR "methylation analysis" OR "methylation sequencing" OR "methylation array" OR "methylation level" OR hypermethylation OR hypomethylation OR "global methylation" OR "genome-wide methylation" OR "methylation biomarker") OR ("histone code" OR histones OR "protein processing post-translational" OR "chromatin assembly and disassembly" OR "histone modification" OR "histone methylation" OR "histone acetylation" OR "histone phosphorylation" OR "histone ubiquitination" OR "histone mark" OR "histone code" OR "histone tail" OR "histone H3" OR "histone H4" OR H3K4me3 OR H3K27ac OR H3K9me3 OR H3K36me3 OR "histone methyltransferase" OR "histone acetyltransferase"

	OR "histone deacetylase" OR HDAC OR HAT OR HMT OR "chromatin modification" OR "chromatin remodeling" OR "epigenetic modification" OR "lysine methylation" OR "lysine acetylation" OR "histone variant") OR ("RNA untranslated" OR microRNAs OR "RNA long noncoding" OR "RNA circular" OR "RNA small interfering" OR "RNA ribosomal" OR "RNA transfer" OR "RNA small nuclear" OR "RNA antisense" OR "non-coding RNA" OR "noncoding RNA" OR ncRNA OR microRNA OR miRNA OR "long non-coding RNA" OR "long noncoding RNA" OR lncRNA OR "circular RNA" OR circRNA OR "small interfering RNA" OR siRNA OR "ribosomal RNA" OR rRNA OR "transfer RNA" OR tRNA OR "small nuclear RNA" OR snRNA OR "small nucleolar RNA" OR snoRNA OR "piwi-interacting RNA" OR piRNA OR "antisense RNA" OR "enhancer RNA" OR "regulatory RNA")) AND ("treatment outcome" OR "patient outcome assessment" OR morbidity OR mortality OR "survival rate" OR "quality of life" OR "physical fitness" OR "exercise tolerance" OR "recovery of function" OR "health status indicators" OR efficiency OR efficacy OR effectiveness OR outcome OR outcomes OR "health outcome" OR "performance outcome" OR "clinical outcome" OR "functional outcome" OR morbidity OR mortality OR survival OR "quality of life" OR QoL OR "physical performance" OR "exercise performance" OR "athletic performance" OR "work performance" OR "cognitive performance" OR recovery OR "functional recovery" OR "symptom severity" OR hospitalization OR rehospitalization OR biomarker OR end point OR "physical function" OR "cognitive function" OR "peak VO2" OR VO2max OR "one-repetition maximum" OR 1RM))
**Refine Type:**	Article, English
**Exclude Type:**	Review Article, Early Access, Editorial Material, Meeting Abstract, Proceeding Paper, Letter, Correction, News ltem, Retracted Publication, Non-English

1.3 Medline

Search	Query
**#1**	(("Exercise"[Mesh] OR "Motor Activity"[Mesh] OR "Physical Exertion "[Mesh] OR "Sports"[Mesh] OR exercise[Title/Abstract] OR "physical activity"[Title/Abstract] OR training[Title/Abstract] OR workout[Title/A bstract]))
**#2**	(("Nutrition Therapy"[Mesh] OR "Diet Therapy"[Mesh] OR "Nutritiona l Support"[Mesh] OR "Dietary Supplements"[Mesh] OR "Food, Formul ated"[Mesh] OR "Health Promotion"[Mesh] OR "Counseling"[Mesh] O R "Nutrition intervention"[Title/Abstract] OR "Dietary intervention"[Titl e/Abstract] OR "Nutritional care"[Title/Abstract] OR "Nutritional suppo rt"[Title/Abstract] OR "Diet therapy"[Title/Abstract] OR "Nutrition ther apy"[Title/Abstract] OR "Dietary modification"[Title/Abstract] OR "Foo d-based intervention"[Title/Abstract] OR Supplementation[Title/Abstract] OR "Medical nutrition therapy"[Title/Abstract] OR "Nutritional counse ling"[Title/Abstract] OR "Dietary management"[Title/Abstract] OR "Die tary advice"[Title/Abstract] OR "Nutrition education"[Title/Abstract]))
**#3**	(("DNA Methylation"[Mesh] OR "CpG Islands"[Mesh] OR "Epigenesis, Genetic"[Mesh] OR "DNA methylation"[Title/Abstract] OR "DNA me thylated"[Title/Abstract] OR "DNA methylome"[Title/Abstract] OR "me thylation pattern"[Title/Abstract] OR "methylation profile"[Title/Abstrac t] OR "methylation status"[Title/Abstract] OR "CpG methylation"[Title/ Abstract] OR "CpG site"[Title/Abstract] OR "CpG island"[Title/Abstrac t] OR "5-methylcytosine"[Title/Abstract] OR "5mC"[Title/Abstract] OR "epigenetic modification"[Title/Abstract] OR "epigenetic marker"[Title/ Abstract] OR methyltransferase[Title/Abstract] OR DNMT[Title/Abstrac t] OR "DNA methyltransferase"[Title/Abstract] OR methylome[Title/Ab stract] OR "methylation analysis"[Title/Abstract] OR "methylation sequ encing"[Title/Abstract] OR "methylation array"[Title/Abstract] OR "met hylation level"[Title/Abstract] OR hypermethylation[Title/Abstract] OR hypomethylation[Title/Abstract] OR "global methylation"[Title/Abstract] OR "genome-wide methylation"[Title/Abstract] OR "methylation biom arker"[Title/Abstract]))

**#4**	(("Histone Code"[Mesh] OR "Histones"[Mesh] OR "Protein Processing, Post-Translational"[Mesh] OR "Epigenesis, Genetic"[Mesh] OR "Chromatin Assembly and Disassembly"[Mesh] OR "Histone modification"[Title/Abstract] OR "Histone methylation"[Title/Abstract] OR "Histone acetylation"[Title/Abstract] OR "Histone phosphorylation"[Title/Abstract] OR "Histone ubiquitination"[Title/Abstract] OR "Histone mark"[Title/Abstract] OR "Histone code"[Title/Abstract] OR "Histone tail"[Title/Abstract] OR "Histone H3"[Title/Abstract] OR "Histone H4"[Title/Abstract] OR H3K4me3[Title/Abstract] OR H3K27ac[Title/Abstract] OR H3K9me3[Title/Abstract] OR H3K36me3[Title/Abstract] OR "Histone methyltransferase"[Title/Abstract] OR "Histone acetyltransferase"[Title/Abstract] OR "Histone deacetylase"[Title/Abstract] OR HDAC[Title/Abstract] OR HAT[Title/Abstract] OR HMT[Title/Abstract] OR "Chromatin modification"[Title/Abstract] OR "Chromatin remodeling"[Title/Abstract] OR "Epigenetic modification"[Title/Abstract] OR "Lysine methylation"[Title/Abstract] OR "Lysine acetylation"[Title/Abstract] OR "Histone variant"[Title/Abstract]))
**#5**	(("RNA, Untranslated"[Mesh] OR "MicroRNAs"[Mesh] OR "RNA, Long Noncoding"[Mesh] OR "RNA, Circular"[Mesh] OR "RNA, Small Interfering"[Mesh] OR "RNA, Ribosomal"[Mesh] OR "RNA, Transfer"[Mesh] OR "RNA, Small Nuclear"[Mesh] OR "RNA, Antisense"[Mesh] OR "Epigenesis, Genetic"[Mesh] OR "Non-coding RNA"[Title/Abstract] OR "Noncoding RNA"[Title/Abstract] OR ncRNA[Title/Abstract] OR "MicroRNA"[Title/Abstract] OR miRNA[Title/Abstract] OR "Long non-coding RNA"[Title/Abstract] OR "Long noncoding RNA"[Title/Abstract] OR lncRNA[Title/Abstract] OR "Circular RNA"[Title/Abstract] OR circRNA[Title/Abstract] OR "Small interfering RNA"[Title/Abstract] OR siRNA[Title/Abstract] OR "Ribosomal RNA"[Title/Abstract] OR rRNA[Title/Abstract] OR "Transfer RNA"[Title/Abstract] OR tRNA[Title/Abstract] OR "Small nuclear RNA"[Title/Abstract] OR snRNA[Title/Abstract] OR "Small nucleolar RNA"[Title/Abstract] OR snoRNA[Title/Abstract] OR "Piwi-interacting RNA"[Title/Abstract] OR piRNA[Title/Abstract] OR "Antisense RNA"[Title/Abstract] OR "Enhancer RNA"[Title/Abstract] OR "Regulatory RNA"[Title/Abstract]))

**#6**	(("Treatment Outcome"[Mesh] OR "Patient Outcome Assessment"[Mesh] OR "Morbidity"[Mesh] OR "Mortality"[Mesh] OR "Survival Rate"[Mesh] OR "Quality of Life"[Mesh] OR "Physical Fitness"[Mesh] OR "Exercise Tolerance"[Mesh] OR "Recovery of Function"[Mesh] OR " Health Status Indicators"[Mesh] OR "Efficiency"[Mesh] OR Efficacy[Title/Abstract] OR Effectiveness[Title/Abstract] OR Outcome[Title/Abstract] OR Outcomes[Title/Abstract] OR "Health outcome"[Title/Abstract] OR "Performance outcome"[Title/Abstract] OR "Clinical outcome"[Title/Abstract] OR "Functional outcome"[Title/Abstract] OR Morbidity[Title/Abstract] OR Mortality[Title/Abstract] OR Survival[Title/Abstract] OR "Quality of life"[Title/Abstract] OR QoL[Title/Abstract] OR "Physical performance"[Title/Abstract] OR "Exercise performance"[Title/Abstract] OR "Athletic performance"[Title/Abstract] OR "Work performance"[Title/Abstract] OR "Cognitive performance"[Title/Abstract] OR Recovery[Title/Abstract] OR "Functional recovery"[Title/Abstract] OR "Symptom severity"[Title/Abstract] OR Hospitalization[Title/Abstract] OR Rehospitalization[Title/Abstract] OR Biomarker[Title/Abstract] OR Endpoint[Title/Abstract] OR "Physical function"[Title/Abstract] OR "Cognitive function"[Title/Abstract] OR "Peak VO2"[Title/Abstract] OR VO2max[Title/Abstract] OR "One-repetition maximum"[Title/Abstract] OR 1RM[Title/Abstract]))

#7	**#7：(#1 AND #2) AND (#3 OR #4 OR #5) AND #6**<br>((("Exercise"[Mesh] OR "Motor Activity"[Mesh] OR "Physical Exertion"[Mesh] OR "Sports"[Mesh] OR exercise[Title/Abstract] OR "physical activity"[Title/Abstract] OR training[Title/Abstract] OR workout[Title/Abstract]) **AND** ("Nutrition Therapy"[Mesh] OR "Diet Therapy"[Mesh] OR "Nutritional Support"[Mesh] OR "Dietary Supplements"[Mesh] OR "Food, Formulated"[Mesh] OR "Health Promotion"[Mesh] OR "Counseling"[Mesh] OR "Nutrition intervention"[Title/Abstract] OR "Dietary intervention"[Title/Abstract] OR "Nutritional care"[Title/Abstract] OR "Nutritional support"[Title/Abstract] OR "Diet therapy"[Title/Abstract] OR "Nutrition therapy"[Title/Abstract] OR "Dietary modification"[Title/Abstract] OR "Food-based intervention"[Title/Abstract] OR Supplementation[Title/Abstract] OR "Medical nutrition therapy"[Title/Abstract] OR "Nutritional counseling"[Title/Abstract] OR "Dietary management"[Title/Abstract] OR "Dietary advice"[Title/Abstract] OR "Nutrition education"[Title/Abstract])) **AND** ((("DNA Methylation"[Mesh] OR "CpG Islands"[Mesh] OR "Epigenesis, Genetic"[Mesh] OR "DNA methylation"[Title/Abstract] OR "DNA methylated"[Title/Abstract] OR "DNA methylome"[Title/Abstract] OR "methylation pattern"[Title/Abstract] OR "methylation profile"[Title/Abstract] OR "methylation status"[Title/Abstract] OR "CpG methylation"[Title/Abstract] OR "CpG site"[Title/Abstract] OR "CpG island"[Title/Abstract] OR "5-methylcytosine"[Title/Abstract] OR "5mC"[Title/Abstract] OR "epigenetic modification"[Title/Abstract] OR "epigenetic marker"[Title/Abstract] OR methyltransferase[Title/Abstract] OR DNMT[Title/Abstract] OR "DNA methyltransferase"[Title/Abstract] OR methylome[Title/Abstract] OR "methylation analysis"[Title/Abstract] OR "methylation sequencing"[Title/Abstract] OR "methylation array"[Title/Abstract] OR "methylation level"[Title/Abstract] OR hypermethylation[Title/Abstract] OR hypomethylation[Title/Abstract] OR "global methylation"[Title/Abstract] OR "genome-wide methylation"[Title/Abstract] OR "methylation biomarker"[Title/Abstract]) **OR** ("Histone Code"[Mesh] OR "Histones"[Mesh] OR "Protein Processing, Post-Translational"[Mesh] OR "Epigenesis, Genetic"[Mesh] OR "Chromatin Assembly and Disassembly"[Mesh] OR "Histone modification"[Title/Abstract] OR "Histone methylation"[Title/Abstract] OR "Histone acetylation"[Title/Abstract] OR "Histone phosphorylation"[Title/Abstract] OR "Histone ubiquitination"[Title/Abstract] OR "Histone mark"[Title/Abstract] OR "Histone code"[Title/Abstract] OR "Histone tail"[Title/Abstract] OR "Histone H3"[Title/Abstract] OR "Histone H4"[Title/Abstract] OR H3K4me3[Title/Abstract] OR H3K27ac[Title/Abstract] OR H3K9me3[Title/Abstract] OR H3K36me3[Title/Abstract] OR "Histone methyltransferase"[Title/Abstract] OR "Histone acetyltransferase"[Title/Abstract] OR "Histone deacetylase"[Title/Abstract] OR HDAC[Title/Abstract] OR HAT[Title/Abstract] OR HMT[Title/Abstract] OR "Chromatin modification"[Title/Abstract

	ct] OR "Chromatin remodeling"[Title/Abstract] OR "Epigenetic modification"[Title/Abstract] OR "Lysine methylation"[Title/Abstract] OR "Lysine acetylation"[Title/Abstract] OR "Histone variant"[Title/Abstract]) **OR** ("RNA, Untranslated"[Mesh] OR "MicroRNAs"[Mesh] OR "RNA, Long Noncoding"[Mesh] OR "RNA, Circular"[Mesh] OR "RNA, Small Interfering"[Mesh] OR "RNA, Ribosomal"[Mesh] OR "RNA, Transfer"[Mesh] OR "RNA, Small Nuclear"[Mesh] OR "RNA, Antisense"[Mesh] OR "Epigenesis, Genetic"[Mesh] OR "Non-coding RNA"[Title/Abstract] OR "Noncoding RNA"[Title/Abstract] OR ncRNA[Title/Abstract] OR "MicroRNA"[Title/Abstract] OR miRNA[Title/Abstract] OR "Long non-coding RNA"[Title/Abstract] OR "Long noncoding RNA"[Title/Abstract] OR lncRNA[Title/Abstract] OR "Circular RNA"[Title/Abstract] OR circRNA[Title/Abstract] OR "Small interfering RNA"[Title/Abstract] OR siRNA[Title/Abstract] OR "Ribosomal RNA"[Title/Abstract] OR rRNA[Title/Abstract] OR "Transfer RNA"[Title/Abstract] OR tRNA[Title/Abstract] OR "Small nuclear RNA"[Title/Abstract] OR snRNA[Title/Abstract] OR "Small nucleolar RNA"[Title/Abstract] OR snoRNA[Title/Abstract] OR "Piwi-interacting RNA"[Title/Abstract] OR piRNA[Title/Abstract] OR "Antisense RNA"[Title/Abstract] OR "Enhancer RNA"[Title/Abstract] OR "Regulatory RNA"[Title/Abstract]))) **AND** ("Treatment Outcome"[Mesh] OR "Patient Outcome Assessment"[Mesh] OR "Morbidity"[Mesh] OR "Mortality"[Mesh] OR "Survival Rate"[Mesh] OR "Quality of Life"[Mesh] OR "Physical Fitness"[Mesh] OR "Exercise Tolerance"[Mesh] OR "Recovery of Function"[Mesh] OR "Health Status Indicators"[Mesh] OR "Efficiency"[Mesh] OR Efficacy[Title/Abstract] OR Effectiveness[Title/Abstract] OR Outcome[Title/Abstract] OR Outcomes[Title/Abstract] OR "Health outcome"[Title/Abstract] OR "Performance outcome"[Title/Abstract] OR "Clinical outcome"[Title/Abstract] OR "Functional outcome"[Title/Abstract] OR Morbidity[Title/Abstract] OR Mortality[Title/Abstract] OR Survival[Title/Abstract] OR "Quality of life"[Title/Abstract] OR QoL[Title/Abstract] OR "Physical performance"[Title/Abstract] OR "Exercise performance"[Title/Abstract] OR "Athletic performance"[Title/Abstract] OR "Work performance"[Title/Abstract] OR "Cognitive performance"[Title/Abstract] OR Recovery[Title/Abstract] OR "Functional recovery"[Title/Abstract] OR "Symptom severity"[Title/Abstract] OR Hospitalization[Title/Abstract] OR Rehospitalization[Title/Abstract] OR Biomarker[Title/Abstract] OR Endpoint[Title/Abstract] OR "Physical function"[Title/Abstract] OR "Cognitive function"[Title/Abstract] OR "Peak VO2"[Title/Abstract] OR VO2max[Title/Abstract] OR "One-repetition maximum"[Title/Abstract] OR 1RM[Title/Abstract]))

**Filters:**	Adaptive Clinical Trial, Classical Article, Clinical Study, Clinical Trial, Comparative Study, Controlled Clinical Trial, Multicenter Study, Obser vational Study, Pragmatic Clinical Trial, Randomized Controlled Trial, English

1.4 Embase

Search	Query
#1	('exercise'/exp OR 'motor activity'/exp OR 'physical exertion'/exp OR ' sport'/exp OR 'exercise':ab,ti OR 'physical activity':ab,ti OR 'training':a b,ti OR 'workout':ab,ti)
#2	('diet therapy'/exp OR 'nutritional support'/exp OR 'dietary supplement' /exp OR 'health promotion'/exp OR 'counseling'/exp OR 'nutrition inte rvention':ab,ti OR 'dietary intervention':ab,ti OR 'nutritional care':ab,ti OR 'nutritional support':ab,ti OR 'diet therapy':ab,ti OR 'nutrition thera py':ab,ti OR 'dietary modification':ab,ti OR 'food-based intervention':a b,ti OR 'supplementation':ab,ti OR 'medical nutrition therapy':ab,ti OR 'nutritional counseling':ab,ti OR 'dietary management':ab,ti OR 'dietar y advice':ab,ti OR 'nutrition education':ab,ti)
#3	('dna methylation'/exp OR 'cpg island'/exp OR 'epigenetics'/exp OR 'd na methylation':ab,ti OR 'dna methylated':ab,ti OR 'dna methylome':ab, ti OR 'methylation pattern':ab,ti OR 'methylation profile':ab,ti OR 'met hylation status':ab,ti OR 'cpg methylation':ab,ti OR 'cpg site':ab,ti OR 'cpg island':ab,ti OR '5-methylcytosine':ab,ti OR '5mc':ab,ti OR 'epigen etic modification':ab,ti OR 'epigenetic marker':ab,ti OR 'methyltransfer ase':ab,ti OR 'dnmt':ab,ti OR 'dna methyltransferase':ab,ti OR 'methylo me':ab,ti OR 'methylation analysis':ab,ti OR 'methylation sequencing':a b,ti OR 'methylation array':ab,ti OR 'methylation level':ab,ti OR 'hyper methylation':ab,ti OR 'hypomethylation':ab,ti OR 'global methylation':a b,ti OR 'genome-wide methylation':ab,ti OR 'methylation biomarker':a b,ti)
#4	('histone code'/exp OR 'histone'/exp OR 'protein processing'/exp OR 'e pigenetics'/exp OR 'chromatin assembly and disassembly'/exp OR 'hist one modification':ab,ti OR 'histone methylation':ab,ti OR 'histone acety lation':ab,ti OR 'histone phosphorylation':ab,ti OR 'histone ubiquitinatio n':ab,ti OR 'histone mark':ab,ti OR 'histone code':ab,ti OR 'histone tail ':ab,ti OR 'histone h3':ab,ti OR 'histone h4':ab,ti OR 'h3k4me3':ab,ti O R 'h3k27ac':ab,ti OR 'h3k9me3':ab,ti OR 'h3k36me3':ab,ti OR 'histone methyltransferase':ab,ti OR 'histone acetyltransferase':ab,ti OR 'histone deacetylase':ab,ti OR 'hdac':ab,ti OR 'hat':ab,ti OR 'hmt':ab,ti OR 'chr omatin modification':ab,ti OR 'chromatin remodeling':ab,ti OR 'epigene tic modification':ab,ti OR 'lysine methylation':ab,ti OR 'lysine acetylati on':ab,ti OR 'histone variant':ab,ti)

**#5**	('untranslated rna'/exp OR 'microrna'/exp OR 'long noncoding rna'/exp OR 'circular rna'/exp OR 'small interfering rna'/exp OR 'ribosomal rna'/exp OR 'transfer rna'/exp OR 'small nuclear rna'/exp OR 'small nucleolar rna'/exp OR 'antisense rna'/exp OR 'epigenetics'/exp OR 'non-coding rna':ab,ti OR 'noncoding rna':ab,ti OR 'ncrna':ab,ti OR 'microrna':ab,ti OR 'mirna':ab,ti OR 'long non-coding rna':ab,ti OR 'long noncoding rna':ab,ti OR 'lncrna':ab,ti OR 'circular rna':ab,ti OR 'circrna':ab,ti OR 'small interfering rna':ab,ti OR 'sirna':ab,ti OR 'ribosomal rna':ab,ti OR 'rrna':ab,ti OR 'transfer rna':ab,ti OR 'trna':ab,ti OR 'small nuclear rna':ab,ti OR 'snrna':ab,ti OR 'small nucleolar rna':ab,ti OR 'snorna':ab,ti OR 'piwi-interacting rna':ab,ti OR 'pirna':ab,ti OR 'antisense rna':ab,ti OR 'enhancer rna':ab,ti OR 'regulatory rna':ab,ti)
**#6**	('treatment outcome'/exp OR 'patient outcome assessment'/exp OR 'morbidity'/exp OR 'mortality'/exp OR 'survival rate'/exp OR 'quality of life'/exp OR 'physical fitness'/exp OR 'exercise tolerance'/exp OR 'functional recovery'/exp OR 'health status indicator'/exp OR 'efficiency'/exp OR 'efficacy':ab,ti OR 'effectiveness':ab,ti OR 'outcome':ab,ti OR 'outcomes':ab,ti OR 'health outcome':ab,ti OR 'performance outcome':ab,ti OR 'clinical outcome':ab,ti OR 'functional outcome':ab,ti OR 'morbidity':ab,ti OR 'mortality':ab,ti OR 'survival':ab,ti OR 'quality of life':ab,ti OR 'qol':ab,ti OR 'physical performance':ab,ti OR 'exercise performance':ab,ti OR 'athletic performance':ab,ti OR 'work performance':ab,ti OR 'cognitive performance':ab,ti OR 'recovery':ab,ti OR 'functional recovery':ab,ti OR 'symptom severity':ab,ti OR 'hospitalization':ab,ti OR 'rehospitalization':ab,ti OR 'biomarker':ab,ti OR 'endpoint':ab,ti OR 'physical function':ab,ti OR 'cognitive function':ab,ti OR 'peak vo2':ab,ti OR 'vo2max':ab,ti OR 'one-repetition maximum':ab,ti OR '1rm':ab,ti)
**#7**	**#7：(#1 AND #2) AND (#3 OR #4 OR #5) AND #6**<br>(('exercise'/exp OR 'motor activity'/exp OR 'physical exertion'/exp OR 'sport'/exp OR 'exercise':ab,ti OR 'physical activity':ab,ti OR 'training':ab,ti OR 'workout':ab,ti) **AND** ('diet therapy'/exp OR 'nutritional support'/exp OR 'dietary supplement'/exp OR 'health promotion'/exp OR 'counseling'/exp OR 'nutrition intervention':ab,ti OR 'dietary intervention':ab,ti OR 'nutritional care':ab,ti OR 'nutritional support':ab,ti OR 'diet therapy':ab,ti OR 'nutrition therapy':ab,ti OR 'dietary modification':ab,ti OR 'food-based intervention':ab,ti OR 'supplementation':ab,ti OR 'medical nutrition therapy':ab,ti OR 'nutritional counseling':ab,ti OR 'dietary management':ab,ti OR 'dietary advice':ab,ti OR 'nutrition education':ab,ti) **AND** (('dna methylation'/exp OR 'cpg island'/exp OR 'epigenetics'/exp OR 'dna methylation':ab,ti OR 'dna methylated':ab,ti OR 'dna methylome':ab,ti OR 'methylation pattern':ab,ti OR 'methylation profile':ab,ti OR 'methylation status':ab,ti OR 'cpg methylation':ab,ti OR 'cpg site':ab,ti OR 'cpg island':ab,ti OR '5-methylcytosine':ab,ti OR '5mc':

	ab,ti OR 'epigenetic modification':ab,ti OR 'epigenetic marker':ab,ti OR 'methyltransferase':ab,ti OR 'dnmt':ab,ti OR 'dna methyltransferase':ab,ti OR 'methylome':ab,ti OR 'methylation analysis':ab,ti OR 'methylation sequencing':ab,ti OR 'methylation array':ab,ti OR 'methylation level':ab,ti OR 'hypermethylation':ab,ti OR 'hypomethylation':ab,ti OR 'global methylation':ab,ti OR 'genome-wide methylation':ab,ti OR 'methylation biomarker':ab,ti) **OR** ('histone code'/exp OR 'histone'/exp OR 'protein processing'/exp OR 'epigenetics'/exp OR 'chromatin assembly and disassembly'/exp OR 'histone modification':ab,ti OR 'histone methylation':ab,ti OR 'histone acetylation':ab,ti OR 'histone phosphorylation':ab,ti OR 'histone ubiquitination':ab,ti OR 'histone mark':ab,ti OR 'histone code':ab,ti OR 'histone tail':ab,ti OR 'histone h3':ab,ti OR 'histone h4':ab,ti OR 'h3k4me3':ab,ti OR 'h3k27ac':ab,ti OR 'h3k9me3':ab,ti OR 'h3k36me3':ab,ti OR 'histone methyltransferase':ab,ti OR 'histone acetyltransferase':ab,ti OR 'histone deacetylase':ab,ti OR 'hdac':ab,ti OR 'hat':ab,ti OR 'hmt':ab,ti OR 'chromatin modification':ab,ti OR 'chromatin remodeling':ab,ti OR 'epigenetic modification':ab,ti OR 'lysine methylation':ab,ti OR 'lysine acetylation':ab,ti OR 'histone variant':ab,ti) **OR** ('untranslated rna'/exp OR 'microrna'/exp OR 'long noncoding rna'/exp OR 'circular rna'/exp OR 'small interfering rna'/exp OR 'ribosomal rna'/exp OR 'transfer rna'/exp OR 'small nuclear rna'/exp OR 'small nucleolar rna'/exp OR 'antisense rna'/exp OR 'epigenetics'/exp OR 'non-coding rna':ab,ti OR 'noncoding rna':ab,ti OR 'ncrna':ab,ti OR 'microrna':ab,ti OR 'mirna':ab,ti OR 'long non-coding rna':ab,ti OR 'long noncoding rna':ab,ti OR 'lncrna':ab,ti OR 'circular rna':ab,ti OR 'circrna':ab,ti OR 'small interfering rna':ab,ti OR 'sirna':ab,ti OR 'ribosomal rna':ab,ti OR 'rrna':ab,ti OR 'transfer rna':ab,ti OR 'trna':ab,ti OR 'small nuclear rna':ab,ti OR 'snrna':ab,ti OR 'small nucleolar rna':ab,ti OR 'snorna':ab,ti OR 'piwi-interacting rna':ab,ti OR 'pirna':ab,ti OR 'antisense rna':ab,ti OR 'enhancer rna':ab,ti OR 'regulatory rna':ab,ti)) **AND** ('treatment outcome'/exp OR 'patient outcome assessment'/exp OR 'morbidity'/exp OR 'mortality'/exp OR 'survival rate'/exp OR 'quality of life'/exp OR 'physical fitness'/exp OR 'exercise tolerance'/exp OR 'functional recovery'/exp OR 'health status indicator'/exp OR 'efficiency'/exp OR 'efficacy':ab,ti OR 'effectiveness':ab,ti OR 'outcome':ab,ti OR 'outcomes':ab,ti OR 'health outcome':ab,ti OR 'performance outcome':ab,ti OR 'clinical outcome':ab,ti OR 'functional outcome':ab,ti OR 'morbidity':ab,ti OR 'mortality':ab,ti OR 'survival':ab,ti OR 'quality of life':ab,ti OR 'qol':ab,ti OR 'physical performance':ab,ti OR 'exercise performance':ab,ti OR 'athletic performance':ab,ti OR 'work performance':ab,ti OR 'cognitive performance':ab,ti OR 'recovery':ab,ti OR 'functional recovery':ab,ti OR 'symptom severity':ab,ti OR 'hospitalization':ab,ti OR 'rehospitalization':ab,ti OR 'biomarker':ab,ti OR 'endpoint':ab,ti OR 'physical function':ab,ti OR 'cognitive function':ab,ti OR 'peak vo2':ab,ti OR 'vo2max

	':ab,ti OR 'one-repetition maximum':ab,ti OR '1rm':ab,ti))
**#8**	#7 AND [embase]/lim NOT ([embase]/lim AND [medline]/lim)

1.5 Scopus

Search	Query
#1	TITLE-ABS-KEY(("exercise" OR "motor activity" OR "physical exertion" OR "sport" OR "training" OR "workout" OR "physical activity") **AND** ("diet therapy" OR "nutritional support" OR "dietary supplement" OR "health promotion" OR "counseling" OR "nutrition intervention" OR "dietary intervention" OR "nutritional care" OR "nutrition therapy" OR "dietary modification" OR "food based intervention" OR "supplementation" OR "medical nutrition therapy" OR "nutritional counseling" OR "dietary management" OR "dietary advice" OR "nutrition education") **AND** (("dna methylation" OR "cpg island" OR "epigenetics" OR "dna methylated" OR "dna methylome" OR "methylation pattern" OR "methylation profile" OR "methylation status" OR "cpg methylation" OR "cpg site" OR "5-methylcytosine" OR "5mc" OR "epigenetic modification" OR "epigenetic marker" OR "methyltransferase" OR "dnmt" OR "dna methyltransferase" OR "methylome" OR "methylation analysis" OR "methylation sequencing" OR "methylation array" OR "methylation level" OR "hypermethylation" OR "hypomethylation" OR "global methylation" OR "genome wide methylation" OR "methylation biomarker") **OR** ("histone code" OR "histone" OR "protein processing" OR "chromatin assembly and disassembly" OR "histone modification" OR "histone methylation" OR "histone acetylation" OR "histone phosphorylation" OR "histone ubiquitination" OR "histone mark" OR "histone tail" OR "histone h3" OR "histone h4" OR "h3k4me3" OR "h3k27ac" OR "h3k9me3" OR "h3k36me3" OR "histone methyltransferase" OR "histone acetyltransferase" OR "histone deacetylase" OR "hdac" OR "hat" OR "hmt" OR "chromatin modification" OR "chromatin remodeling" OR "lysine methylation" OR "lysine acetylation" OR "histone variant") **OR** ("untranslated rna" OR "microrna" OR "long noncoding rna" OR "circular rna" OR "small interfering rna" OR "ribosomal rna" OR "transfer rna" OR "small nuclear rna" OR "small nucleolar rna" OR "antisense rna" OR "non coding rna" OR "noncoding rna" OR "ncrna" OR "mirna" OR "lncrna" OR "circrna" OR "sirna" OR "rrna" OR "trna" OR "snrna" OR "snorna" OR "pirna" OR "enhancer rna" OR "regulatory rna")) **AND** ("treatment outcome" OR "patient outcome assessment" OR "morbidity" OR "mortality" OR "survival rate" OR "quality of life" OR "physical fitness" OR "exercise tolerance" OR "functional recovery" OR "health status indicator" OR "efficiency" OR "efficacy" OR "effectiveness" OR "outcome" OR "outcomes" OR "health outcome" OR "performance outcome" OR "clinical outcome" OR "functional outcome" OR "survival" OR "qol" OR "physical performance" OR "exercise performance" OR "athletic performance" OR "work performance" OR "cognitive performance" OR "recovery" OR "symptom severity" OR "hospitalization" OR "rehospitalization" OR "biomarker" OR "endpoint" OR "physical

	function" OR "cognitive function" OR "peak vo2" OR "vo2max" OR "one repetition maximum" OR "1rm"))
**Refine Type:**	Article, Journal, English
**Exclude Type:**	Article in press, Book series, Conference paper, Review, Conference review, Note,Book chapter, Letter,Erratum, Editorial, Systematic Review, Review, Meta Analysis
