# Supplementary material for: Exercise and nutrition as epigenetic regulators of gene expression: an exploratory scoping review with bibliometric analysis
Source: Front Nutr. 2026 Mar 10;13:1773920. doi: 10.3389/fnut.2026.1773920 (PMC13008672; doi:10.3389/fnut.2026.1773920)
Supplement: Supplementary file 3 [file Data_Sheet_3.pdf]

- Appendix 3 provides a descriptive overview of the epigenetic mechanisms directly examined in the included studies. The classifications presented in Table S3 are based on the epigenetic markers explicitly measured and reported in each study and are intended to support evidence mapping rather than mechanistic or causal inference.

Appendix 3

Table S3. Table S3. Overview of epigenetic-related biological themes reported in studies of combined interventions of exercise and nutrition

Research sources	DNA Methylation	Histone Modification	Non-Coding RNA
Hilder et al.(2019)	✓[1]	–	–
Bischoff-Ferrari et al.(2025)	✓[2]	–	–
Aminizadeh et al.(2023)	–	–	✓[8]
Hunter et al.(2019)	✓[3]	–	–
Heianza et al.(2022)	–	–	✓[10]
Fiorito et al.(2021)	✓[4]	–	–
Dani et al.(2021)	–	✓[6]	–
Aida et al.(2025)	✓[5]	–	–
Schwarz et al.(2019)	–	–	✓[11]
D'Souza et al.(2019)	–	–	✓[12]
Parr et al.(2016)	–	–	✓[13]
Pastuszak-Lewandoska et al.(2019)	–	–	✓[14]
Morikawa et al.(2018)	✓[5]	–	–
Martins et al.(2020)	–	✓[7]	–
Margolis et al.(2017)	–	–	✓[15]
Margolis et al.(2022)	–	–	✓[16]
Tang Donghui et al.(2019)	–	–	✓[17]

Note: "✓" indicates that the corresponding epigenetic marker or process was directly measured and reported in the study; "–" indicates that it was not assessed.

Note (Further explanation and clarification)

(1) DNA Methylation

1. **Whole-genome methylation analysis (Illumina EPIC):** Identify differentially methylated regions (DMRs) across the entire genome, discover changes in methylation levels of genes such as IRF2, and enrich DMRs in pathways such as cell adhesion and PI3K/AKT.
2. **DNA methylation aging clocks (DNAmAge Clocks):** Models that estimate biological age based on DNA methylation levels, assessing PC-PhenoAge, PC-GrimAge, GrimAge2 (second-generation clock), DunedinPACE (third-generation clock, reflecting aging rate), and DNAm plasma proteins (such as PAI-1, Leptin).
3. **Whole-genome and gene-specific methylation:** The LUMA technology is used to assess the level of whole-genome methylation, and pyrosequencing is employed for high-precision methylation analysis of specific genes such as PPARGC1A and IL6.
4. **Epigenetic aging and stability:** Analyzing DNAmGrimAge acceleration (DNAmGrimAA) and epigenetic mutational load (EML), the latter referring to the sum of abnormal methylation events occurring in the genome.
5. **Gene promoter methylation (pyrosequencing):** High-precision quantification of methylation at specific CpG sites in the promoter region of the NFKB2 gene.

(2) Histone Modification

6. **Global histone acetylation (colorimetric assay):** This assay measures the overall acetylation levels of histone H3 and H4, reflecting the transcriptional activity of chromatin.
7. **Global histone H4 acetylation (colorimetric assay):** specifically detects the overall acetylation level of histone H4.

(3) Non-Coding RNA

8. **Serum miRNA expression (RT-qPCR):** Quantitative analysis of the expression levels of specific miRNAs (such as miR-155, -19b, -146a) in the circulation.
9. **DNA methyltransferase (DNMT) mRNA expression:** Detect the expression levels of genes encoding key DNA methylation enzymes (DNMT1, DNMT3a, DNMT3b).
10. **Serum miRNA expression (next-generation sequencing):** Analyze the miRNA expression profile in serum without bias, with a focus on the miR-99/100 family.
11. **Skeletal muscle miRNA expression (RT-qPCR):** Analyze the basal expression levels of miRNAs (such as miR-23a, -23b) in muscle tissue.
12. **Skeletal muscle miRNA expression (RT-PCR):** Detecting acute expression changes of multiple miRNAs in skeletal muscle after resistance exercise.
13. **Circulating miRNA (c-miRNA) expression (customized PCR array):** A customized high-throughput method is used to analyze a pre-selected set of miRNAs in plasma.
14. **Inflammation-related miRNA and mRNA expression (qRT-PCR):** Simultaneously analyze the expression of inflammation-related miRNAs and their potential target gene mRNAs in peripheral blood
15. **Skeletal muscle myogenic miRNA (myomiR) expression (RT-qPCR):** Quantitative analysis of the expression of muscle-specific miRNAs, such as miR-1, -206, -208a, and -499.
16. **Skeletal muscle miRNA expression (RT-qPCR microarray):** A medium-throughput screening was conducted using a preset panel containing 84 miRNAs.
17. **Serum miR-126 expression (qPCR):** specifically quantifies the expression level of miR-126 in serum.
