# Supplementary material for: Exercise and nutrition as epigenetic regulators of gene expression: an exploratory scoping review with bibliometric analysis
Source: Front Nutr. 2026 Mar 10;13:1773920. doi: 10.3389/fnut.2026.1773920 (PMC13008672; doi:10.3389/fnut.2026.1773920)
Supplement: Supplementary file 4 [file Data_Sheet_4.pdf]

- Appendix 4 presents descriptive summaries of the design characteristics, intervention components, epigenetic measurements, and reported findings of the included studies. These summaries are intended to provide contextual detail in support of the scoping review and reflect reported associations within individual studies, rather than establishing causal relationships or definitive mechanistic conclusions.

Appendix 4

Table S4. Summary of the study characteristics (N=17)

Author (Year, Country)	Design	Population	Intervention	Cycle and Follow-up	Mechanistic Exploration	Findings and Conclusions
Hibler et al. (2019), United States	Randomized controlled trial	**1. N=68;**<br><br>**2. Traits:** Adults aged 18-65 with unhealthy lifestyle (insufficient intake of fruits/vegetables, high intake of saturated fat, excessive leisure screen time, insufficient moderate-to-high intensity physical activity)	**Study Protocol:** 3 groups of random control.<br><br>**1. Simultaneous intervention group:** Exercise (increase moderate-to-high intensity physical activity to ≥150 minutes/week)<br><br>**2. Sequential intervention group:** nutrition intervention first, then exercise intervention<br><br>3. **Control group:** No exercise nutrition intervention (received sleep and stress management guidance)	**1. Intervention duration:** 12 weeks<br><br>**2. Follow-up and Measurement:** baseline, 3 months, 9 months	**1. Technology:** Illumina EPIC (850k) whole genome methylation.<br><br>**2. Analytical indicator:** Differentially methylated regions (DMRs).	**1. Methylation changes:** Compared with the control group, 154 and 298 differential methylation regions were found in the intervention group at 3 months and 9 months respectively, and more than 60% of the regions showed decreased methylation levels.<br><br>**2. Key gene:** IRF2 gene region showed decreased methylation at 3 months and 9 months.<br><br>**3. Pathway** enrichment: Differentially methylated regions were enriched in pathways related to immunity, cell cycle, and carcinogenesis, such as cell adhesion, PI3K/AKT, and Wnt/β-catenin.<br><br>**Conclusion:** This study reported that combined interventions of exercise and dietary were associated with changes in DNA methylation patterns of genes related to chronic disease risk within the study population.
Bischoff-Ferrari et al. (2025), Switzerland (multinational collaboration)	Multicenter, randomized, double-blind, placebo-controlled trial (2×2×2 factorial design)	**1. N=777;**<br>**2. Traits:** Relatively healthy older adults in the community (average age 75.5 years, 59.7% women, 52.3% meeting the criteria for healthy ageing)	**Study Protocol:** 2×2×2 factorial design (8 combinations).<br><br>**1. Exercise intervention:** Home strength training (3 times a week, 30 minutes per session) or health education (no exercise).<br><br>**2. Nutritional intervention:** Vitamin D (2000 IU/day) or placebo; Omega-3 (1 g/day) or placebo.<br><br>**3. Combination example:**<br>① Vitamin D+Omega-3+Strength Training<br><br>② Vitamin D+Omega-3+Health education<br><br>③ Vitamin D+Omega-3 placebo+strength training<br><br>④ Vitamin D+Omega-3 placebo+health education<br><br>⑤ Vitamin D placebo+Omega-3+strength training<br><br>⑥ Vitamin D placebo + Omega-3 + health education<br><br>⑦ Vitamin D placebo + Omega-3 placebo + strength training<br><br>⑧ Vitamin D placebo + Omega-3 placebo + health education	**1. Intervention duration:** 3 years<br><br>**2. Follow-up and Measurement:** Baseline, third years<br><br>**3. Samples:** Whole blood was collected at baseline and 3 years for DNA methylation analysis.	**1. Technique:** Whole genome DNA methylation assay.<br><br>**2. Analysis metrics:**<br>① Second-generation clock: PC-PhenoAge,PC-GrimAge,GrimAge2<br>② Third-generation clock**:** DunedinPACE (aging rate)<br>③ DNA replacement: GrimAge-associated plasma proteins (e.g., PAI-1, Leptin, TIMP-1).	**1. Primary effect:** Daily Omega-3 supplementation can slow the progression of multiple DNA methylation-related aging clock markers, with effect sizes ranging from 0.16 to 0.32, equivalent to delaying biological aging by 2.9 to 3.8 months.<br><br>**2. Combined benefits:** On the PC-PhenoAge clock, combined interventions of Omega-3 with vitamin D and/or exercise showed cumulative benefits; multiple combination regimens also demonstrated cumulative benefits for aging-related markers such as DNA methylation PAI-1 and B2M.<br><br>**3. Individual differences:** Individuals with lower baseline Omega-3 levels or adequate vitamin D intake benefited more from the intervention.<br><br>**Conclusion:** This trial reported that omega-3 supplementation was associated with favorable changes in DNA methylation–based aging indicators, and that combined interventions including vitamin D and exercise were discussed in relation to cumulative patterns of change in selected epigenetic aging markers within the trial context.

Author (Year, Country)	Design	Population	Intervention	Cycle and Follow-up	Mechanistic Exploration	Findings and Conclusions
**Aminizadeh et al.** (2023), Iran, United States	Randomized, double-blind, placebo-controlled trial	**1. N=32；**<br>**2. Traits:** Male professional cyclists (mean age 25.6±3.8 years, healthy)	**Study Protocol:** 4 groups of random control.<br>① Placebo group: no exercise + starch placebo<br>② Exercise group (EX): Exercise (bicycle ergometer, 3 times/week)<br>③ MitoQ group: No exercise + MitoQ supplementation (20 mg/day)<br>④ Exercise + MitoQ group: Exercise + MitoQ supplementation	**1. Intervention duration:** 4 weeks;<br>**2. Follow-up and Measurement:** Blood samples were collected at baseline and 30 days after the intervention.<br>**3. Sample:** Serum was used to analyze ROS, antioxidant enzymes, and miRNA.	**1. Technique:** RT-qPCR;<br>**2. Analytical indicators:** The expression levels of miRNA-155, -181b, -19b, and-146a in serum	**1. Oxidative stress:** the serum reactive oxygen species level was decreased in the exercise combined with MitoQ group and the exercise alone group, the glutathione peroxidase level was increased in all the intervention groups, and the superoxide dismutase level was unchanged.<br>**2. miRNA expression:** Compared with the exercise-only group, the exercise combined with MitoQ group showed decreased expression of miRNA-155 and miRNA-19b, and increased expression of miRNA-146a.<br>**Conclusion:** The study reported that exercise combined with MitoQ supplementation was associated with changes in oxidative stress markers and circulating miRNA expression, alongside observed alterations in vascular-related outcomes in professional athletes.
Hunter et al. (2019), Britain	Randomized, double-blind, repeated measurement crossover trial	**1. N=8;**<br>2. **Traits**: Healthy male professional cyclists (mean age 39.5±5.9 years)	**Study Protocol:** Randomized crossover design<br>① n-3 PUFA phase: Exercise (1-hour cycling) + Nutrition (5.7g/day of n-3 PUFA supplementation)<br>② Extra Virgin Olive Oil (EVOO) Phase: Exercise (same regimen as above) + Nutrition (supplement with 6g of extra virgin olive oil daily)<br>③ The two stages were separated by a 4-week washout period.	**1. Intervention duration:** 4 weeks;<br>**2. Follow-up and Measurement:** Baseline, immediate sampling after intervention (blood sample)	**1. Techniques:** LUMA (whole-genome methylation) and pyrosequencing (gene-specific);<br>**2. Analytical indicators:** global methylation, methylation of PPARGC1A, IL6, TNF genes, and DNMT mRNA expression	**1. Methylation changes:** Acute aerobic exercise induced whole genome hypomethylation and PPARGC1A gene-specific hypomethylation. n-3 polyunsaturated fatty acids and extra virgin olive oil had opposite effects on methylation of CpG sites of IL6 gene.<br>**2. Gene expression:** The mRNA expression of PPARGC1A was increased after exercise; the mRNA expression of DNMT3a and DNMT3b was decreased after exercise, and the mRNA expression of DNMT1 was decreased after consumption of extra virgin olive oil.<br>**3. Key association:** The methylation level of PPARGC1A gene is associated with exercise performance and oxidative stress markers.<br>**Conclusion:** This study reported that acute aerobic exercise combined with different nutritional fat sources was associated with changes in DNA methylation levels of metabolism- and inflammation-related genes in leukocytes, occurring alongside alterations in DNA methyltransferase expression.

Author (Year, Country)	Design	Population	Intervention	Cycle and Follow-up	Mechanistic Exploration	Findings and Conclusions
Heianza et al. (2022), a multinational collaboration involving the United States, Israel, Germany, and other countries	Central trial	**1. N=144;**<br><br>**2. Traits:** Adults with abdominal obesity (mean age of 48 years, men account for ~88%)	**Study Protocol:** Multicenter randomized trial<br><br>① Basic intervention (diet): LF group (low-fat, high-fiber diet) or MED/LC group (Mediterranean/low-carbohydrate diet)<br><br>② Overlapping intervention (exercise): After 6 months, participants were randomly assigned to either the increased physical activity group or the maintenance group, with the intervention lasting for 12 months.	**1. Intervention duration:** 18 months;<br><br>**2. Follow-up and Measurement:** baseline, 6 months, 18 months (blood sample collection and body composition assessment by MRI)	**1. Technology:** Next-generation sequencing (Illumina);<br><br>**2. Analytical indicators:** Serum expression levels of miR-99a-5p, miR-99b-5p, and miR-100-5p	**1. miRNA alterations** and adipose reduction: Decreased expression levels of miR-100-5p and miR-99a-5p in circulation were associated with reduced visceral fat, subcutaneous deep/epidermal fat, total fat mass, and intrahepatic fat percentage.<br><br>**2. Pancreatic fat and metabolic improvement:** The decrease of miR-100-5p and miR-99b-5p was associated with the reduction of pancreatic fat, the decrease of fasting blood glucose and the improvement of β-cell function.<br><br>**3. Multiple ectopic fat:** The decrease of miR-100-5p was also associated with the decrease of renal parenchymal fat, renal sinus fat, intermuscular fat and pericardial fat.<br><br>**Conclusion:** The findings indicated that lifestyle interventions combining dietary modification and physical activity were associated with changes in circulating miR-99/100 family expression, occurring in parallel with reductions in visceral and ectopic fat depots and improvements in glycemic-related measures.
Fiorito et al. (2021), Italy	A 24-month randomized factorial intervention trial (DAMA study)	**1. N=219;**<br><br>**2. Traits:** Healthy postmenopausal women (50-69 years)	**Study Protocol:** 2×2 factorial intervention<br>① Combined intervention group: Exercise (increased moderate-to-high intensity activity) + Nutrition (Mediterranean diet)<br><br>② Single exercise group: only exercise intervention<br><br>③ Single diet group: only nutrition intervention<br><br>④ Control group: no specific intervention	**1. Intervention duration:** 24 months;<br><br>**2. Follow-up and Measurement:** Blood samples were collected at baseline and at the end of the intervention (24 months) for whole genome DNA methylation analysis	**1. Technology:** Illumina HumanMethylation450 BeadChip;<br><br>**2. Analytical metrics:** DNAmGrimAge acceleration (DNAmGrimAA) and epigenetic mutation load (EML)	**1. Slowing of the epigenetic clock:** Lifestyle interventions combined with the study slowed the progression of DNA methylation GrimAge.<br><br>**2. Enhanced epigenomic stability:** The intervention reduced the epigenetic mutation burden.<br><br>**3. Mechanism complementarity: T**he intervention effect is achieved by regulating DNA methylation associated with metabolism and inflammation, which replaces plasma proteins (e.g., PAI-1, Leptin), and by reversing epigenetic mutations enriched in cancer pathways (e.g., Wnt, Hippo).<br><br>**Conclusion:** This study reported that long-term combined exercise and dietary interventions were associated with slower changes in DNA methylation–based aging indicators and measures of epigenomic stability within the study population.

Author<br>(Year, Country)	Design	Population	Intervention	Cycle and Follow-up	Mechanistic Exploration	Findings and Conclusions
Dani et al. (2021), Brazil	Double-blind randomized controlled trial	1. **N=29;**<br><br>2. **Traits:** Healthy older women (≥59 years)	**Study Protocol:** 3 groups of random control (no independent control group).<br><br>① GJG group: Nutritional supplementation (daily intake of 400 mL red grape juice).<br><br>② PLEG group: Exercise (60 minutes twice weekly) + Nutrition (placebo).<br><br>③ GJEG group: Exercise (same as PLEG group) + Nutrition (red grape juice).	1. **Intervention duration:** 4 weeks;<br><br>2. **Follow-up and Measurement:** Blood samples were collected at baseline and after the intervention.	1. **Technique:** Colorimetry;<br><br>2. **Analytical metrics:** Histone H3 and H4 global acetylation in peripheral blood mononuclear cells	1. **Epigenetic changes:** The global acetylation levels of histone H3 and H4 in all groups were unchanged after intervention.<br><br>2. **Inflammatory markers:** The level of interleukin-6 in the exercise group was decreased.<br><br>3. **Oxidative stress:** The carbonyl level of protein (oxidative damage) and the activity of superoxide dismutase (antioxidant enzyme) were increased in all groups, but the non-enzymatic antioxidant defense (thiol group) was increased only in red grape juice group.<br><br>**Conclusion:** The study reported that exercise and grape juice supplementation were associated with changes in oxidative stress and inflammatory markers, while no consistent alterations in global histone acetylation were observed following the interventions.
Aida et al. (2025), Japan	Randomized, double-blind, placebo controlled trial	1. **N=51；**<br><br>2. **Traits:** Older adults of approximately 70 years of age who have undergone ≥6 months of intermittent walking training but still have hyperglycemia (fasting blood glucose>110 mg/dl or HbA1c>6.0%)	**Study Protocol:** 2 groups of random control.<br><br>① CNT group: Exercise (intermittent walking training) + Nutrition (daily intake of 150g white rice).<br><br>②HPR group: Exercise (intermittent walking training) + Nutrition (daily intake of 150g high-pressure processed rice).	1. **Intervention duration:** 4 months;<br><br>2. **Follow-up and Measurement:** Blood samples were collected at baseline and after the intervention, and continuous glucose monitoring (CGM) was performed.	1. **Technique:** Pyrosequencing;<br><br>2. **Analytical indicators:** DNA methylation level of CpG sites (1-6) in the promoter region of NFKB2 gene	1. **Improvement of blood glucose control:** The area under the curve of blood glucose after breakfast in the high-pressure rice group was decreased, and the blood glucose variability was improved.<br><br>2. **Methylation change:** The methylation of NFKB2 gene decreased in the control group, but the decrease was inhibited in the high-pressure rice group, and there was a significant difference between the two groups.<br><br>3. **Correlation:** The change of NFKB2 gene methylation level was negatively correlated with the change of the area under the curve of blood glucose after breakfast.<br><br>**Conclusion:** This study reported that combined exercise and dietary interventions were associated with improvements in glycemic profiles and concurrent changes in NFKB2 DNA methylation, suggesting a potential epigenetic link within the specific intervention setting.

Author (Year, Country)	Design	Population	Intervention	Cycle and Follow-up	Mechanistic Exploration	Findings and Conclusions
Schwarz et al. (2019), United States*	Randomized, double-blind, placebo-controlled trial (parallel design)	1. **N=16;**<br><br>2. **Traits:** healthy, recreational male (average age of 22.5 years, with experience in resistance training)	**Study Protocol:** 2 groups of random control.<br><br>① Exercise intervention (general): 4 weeks of structured resistance training (4 days per week)<br><br>② BMB experimental group: Take 26.1g compound supplement before training<br><br>③ Placebo control group: Took the same amount of placebo before training, which had similar appearance and taste.	1. **Intervention duration:** 4 weeks;<br><br>2. **Follow-up and Measurement:** Testing at baseline and after intervention (body composition, strength, blood, muscle biopsy)	1. **Technique:** RT-qPCR;<br><br>2. **Analytical indicators:** Expression levels of miR-126, -23b, -16, -23a, and-15a in skeletal muscle	1. **Body composition:** The BMB group showed greater increases in total body weight and lean body mass compared to the placebo group.<br><br>2. **Maximum strength:** The BMB group showed a greater increase in 1RM for squats than the placebo group. Both groups demonstrated improvements in 1RM for bench press, but the differences were statistically insignificant.<br><br>3. **microRNA expression:** Resistance training increased the baseline expression levels of miR-23a and miR-23b in skeletal muscle, but the supplement did not cause intergroup differences.<br><br>4. **4Serum IGF-1 and safety:** No changes in serum IGF-1 levels in both groups; BMB supplementation did not adversely affect resting hemodynamics or clinical blood safety indicators.<br><br>**Conclusion:** The findings indicated that resistance training combined with nutritional supplementation was associated with changes in skeletal muscle miRNA expression and improvements in strength- and body composition–related outcomes within the study context.
D'Souza et al. (2019), New Zealand	Randomized,placebo controlled trial (three groups in parallel design)	1. **N=23;**<br><br>2. **Participants:** healthy elderly men (mean age 67.9±0.9 years), with no history of regular resistance training and no regular activity level.	**Study Protocol:** single exercise, 3 groups of random control.<br><br>① Exercise intervention (generalized): Single resistance exercise (3 sets, 8-10 reps, 80% 1RM)<br><br>② Placebo g roup: Non-caloric placebo after exercise<br><br>③ 20g PROTEOM: 20g WHEY PROTEIN AFTER EXERCISE<br><br>④ 40g PROTEOGEN: 40g of whey protein after exercise	1. **Intervention duration:** Single exercise + single nutritional supplement;<br><br>1. **Follow-up and Measurement:** Muscle biopsies were obtained before exercise (baseline), 2 hours and 4 hours after exercise.	2. **Technique:** RT-PCR;<br><br>3. **Analytical indicators:** The expression levels of 19 **miRNAs** (including miR-1, -15a, -16, -99a, -148b, -149, -451a, -499a, etc.) in skeletal muscle were analyzed, and their correlation with the phosphorylation of p-AktSer473 and p-P70S6KTh389 was also investigated.	1. **miRNA expression differences:** Compared with the placebo group, the expression of miR-15a, -99a, -148b, -149, and-499a was suppressed in the 20g and 40g protein groups 4 hours post-exercise, while miR-1 was only suppressed in the 40g group.<br><br>2. **Specificity:** miR-451a expression was elevated only in 40g protein samples at 2 and 4 hours post-exercise.<br><br>3. **Signal pathway association**: miR-208a and miR-499a expression changes were strongly positively correlated with the phosphorylation level of p-P70S6KTh389, and miR-206 and miR-208a expression changes were correlated with the phosphorylation level of p-AktSer473.<br><br>4. **Muscle mass correlation:** The expression level of miR-133a in resting state was negatively correlated with the cross-sectional area of thigh.<br><br>**Conclusion:** This study reported that whey protein supplementation following resistance exercise was associated with acute changes in skeletal muscle miRNA expression, occurring alongside alterations in anabolic signaling markers in older men.

Author (Year, Country)	Design	Population	Intervention	Cycle and Follow-up	Mechanistic Exploration	Findings and Conclusions
**Parr et al.** (2016), Australia	Multicenter, randomized, parallel controlled intervention trial	1. **N=40;** 2. **Participants:** Overweight/obese adults (mean age 48±6 years) were divided into a high response group (HiRes,>10%) and a low response group (LoRes, <5%) based on weight loss after 16 weeks of intervention.	**Study Protocol:** A study of responders under a uniform energy limit ① Exercise intervention: about 250 kcal are consumed through exercise every day; ② Nutritional intervention: Daily dietary energy intake was limited to approximately 250 kcal, and participants were randomly assigned to one of three dietary groups with different macronutrient compositions.	1. **Intervention duration:** 16 weeks; 2. **Follow-up and Measurement:** Fasting plasma samples were collected at baseline and at 16 weeks after intervention.	1. **Technology:** Customized 96-well miScript miRNA PCR array; 2. **Analytical metrics:** Relative expression levels of 13 preselected circulating microRNAs (c-miRNAs)	1. **Body weight and body composition:** The HiRes group showed greater weight and fat reduction than the LoRes group. 2. **miRNA expression profiles:** baseline levels showed higher c-miR-935 expression in the low-resolution (LoRes) group than in the high-resolution (HiRes) group. After intervention, c-miR-221-3p and c-miR-223-3p were upregulated in both groups, while c-miR-140 was elevated exclusively in the LoRes group. **Conclusion:** The study reported that circulating miRNA expression profiles were associated with individual variability in weight loss responses following combined dietary and exercise interventions, suggesting their potential relevance as responsive molecular signals.
**Pastuszak-Lewandoska et al.** (2019), Poland, South Africa	Randomized, double-blind, placebo-controlled trial (two-group parallel design)	1. **N=20;** 2. **Traits:** Healthy amateur male ultramarathon runners (average age of about 40 years)	**Study Protocol:** Randomised controlled trial; 1. **Exercise intervention (general):** a single 100 km ultramarathon; 2. **Nutrition interventions:** ① Supplement group: oral administration of 10,000IU vitamin D_3 daily for 2 weeks; ② Control group: no vitamin D supplementation.	1. **Intervention duration:** 2 weeks of nutritional supplementation, the end point was 100 km run; 2. **Follow-up and Measurement:** Blood samples were collected before the 100 km run (baseline) and 12 hours after the run.	1. **Technique:** Real-time quantitative PCR; 2. **Analytical indicators:** The abundance of six inflammation-related miRNAs (miR-21, -146a, -150, -155, -222, -223) in peripheral blood, and the mRNA expression levels of seven inflammatory factors/VDR.	1. **Gene and miRNA expression:** In the vitamin D supplementation group, the expression of miR-155 and miR-223 was up-regulated, while the expression of miR-155 and miR-223 was down-regulated. 2. **Correlation:** At baseline, both groups showed a negative correlation between IL-6 mRNA levels and miR-155 or miR-223 abundance. **Conclusion:** This study reported that vitamin D supplementation in the context of extreme endurance exercise was associated with changes in inflammatory gene expression and circulating miRNA profiles during exercise-induced immune responses..
**Morikawa et al.** (2018), Japan	Randomized, single-blind, placebo controlled trial	1. **N=30;** 2. **Traits:** Healthy elderly women (mean age of 65 years) who have completed>6 months of intermittent walking training and are at the plateau of training effect	**Study Protocol:** 2 random control groups 1. **Exercise intervention (generalized):** intermittent walking training (≥4 days weekly, ≥5 sets daily); 2. **Nutrition interventions:** ① Tofu dry group: immediately after training, consume tofu dry (111 kcal,9.6g protein) ② Placebo group: Take an equal amount of placebo (108 kcal,0.2g protein) immediately after training	1. **Intervention duration:** 5 months; 2. **Follow-up and Measurement:** Whole blood samples were collected at baseline and 5 months after the intervention to measure body composition and muscle strength.	1. **Technique:** Pyrosequencing; 2. **Analytical indicators:** Analysis of the DNA methylation level of six CpG sites in the promoter region of NFKB2 gene	1. **Methylation changes:** Compared with the placebo group, the dry tofu group showed higher methylation levels at six CpG sites in the NFKB2 promoter region, with an average increase of 18% (P=0.035). 2. **Muscle and strength:** The cross-sectional area of thigh muscles increased in both groups, but there was no difference in the increment of muscle strength and muscle mass between the two groups. **Conclusion:** The findings indicated that soy protein supplementation following long-term walking training was associated with changes in NFKB2 DNA methylation and inflammatory-related markers within the study population.

Author (Year, Country)	Design	Population	Intervention	Cycle and Follow-up	Mechanistic Exploration	Findings and Conclusions
Martins et al. (2020), Brazil*	Randomized, double blind, placebo controlled crossover trial	**1. N=12;**<br><br>**2. Traits:** Male volleyball players (16.5±0.6 years old) had high competitive level and high training load.	**Study Protocol:** 3 condition cross (control WB, grape juice GJ, placebo PLA).<br><br>① GJ protocol: Exercise combined with nutrition (400mL grape juice daily for 14 days)<br><br>②PLA condition: Exercise + nutrition (equivalent placebo without polyphenols)<br><br>③ WB condition: Only exercise, no nutritional supplementation	**1. Intervention duration:** 14 days;<br><br>**2. Follow-up and Measurement:** Baseline, blood collection immediately after the event;<br><br>**3. Sample:** Blood samples were analyzed for oxidative stress, inflammation, and histone H4 acetylation.	**1. Technique:** Colorimetry;<br><br>**2. Analytical parameter:** Global histone H4 acetylation level (ng/mg).	**1.** Oxidative stress: The lipid peroxidation (MDA) and DNA damage were decreased in GJ group after exercise.<br><br>**2.** Inflammation and muscle damage: No elevation of IFN-γ, IL-4 or CK-NAC was observed in the GJ group.<br><br>**3.** Epigenetics: The H4 acetylation level decreased in WB group after exercise, but no changes were observed in GJ and PLA groups.<br><br>**Conclusion:** This study reported that grape juice supplementation during training was associated with changes in oxidative stress and DNA damage markers, while no consistent alterations in histone H4 acetylation were observed following the intervention.
Margolis et al. (2017), United States	Randomized, double-blind, placebo-controlled trial (2×2 factorial design)	**1. N=25;**<br><br>**2. Traits:** Healthy adults (18-39 years of age), in good physical condition, with a BMI of 22–29 kg/m².	**Study Protocol:** 4 groups of random control.<br><br>① LC/EAA+CHO: Weight-bearing endurance training combined with nutritional supplementation (10g essential amino acids + 46g carbohydrates).<br><br>② CE/EAA+CHO: Cycling endurance training plus nutrition (as above).<br><br>③ Group C: Exercise combined with no nutritional supplementation (placebo).	**1. Intervention duration:** A single 90-minute exercise session;<br><br>**2. Follow-up and Measurement:** Baseline, immediately after exercise, 3 hours after recovery;<br><br>**3. Sample:** Muscle biopsy.	**1. Technique:** RT-qPCR;<br><br>**2. Analytical indicators:** Expression of myomiRs (miR-1-3p, -206, -208a-5p, -499, etc.) in skeletal muscle.	**1. MiR expression:** weight-bearing exercise (LC) down-regulated the expression of miR-1-3p, -206, -208a-5p, and-499; EAA+CHO further inhibited their expression.<br><br>**2. Association with MPS:** miR-206 and miR-499 expression showed negative correlation with post-exercise muscle protein synthesis rate.<br><br>**Conclusion:** The study reported that different exercise modalities combined with post-exercise nutritional supplementation were associated with changes in skeletal muscle miRNA expression, occurring alongside alterations in muscle protein synthesis–related signaling.
Margolis et al. (2022), United States	Randomized, crossover, controlled trials	**1. N=7;**<br><br>**2. Traits:** The subjects were healthy males (21±3 years old) with a mean body weight of 83±13 kg and a peak oxygen uptake of 43±2 mL/kg/min.	**Study Protocol:** 2 stage cross over<br><br>① Exercise intervention (whole body): single session of cycling to exhaustion<br><br>② CHO phase: During the recovery period, carbohydrate intake was maintained at 1g/kg/h.<br><br>③ The recovery period: consume the same amount of non-nutritive control beverage	**1. Intervention duration:** Single exercise+ 3 hours of recovery;<br><br>**2. Follow-up and Measurement:** Baseline fasting, at the end of 3 hours of recovery;<br><br>**3. Sample:** Muscle biopsy (lateral femoral muscle).	**1. Technique:** RT-qPCR microarray analysis (84 miRNAs);<br><br>**2. Analytical indicators:** Differentially expressed miRNAs in skeletal muscle (e.g., Let7i-5p, miR-195-5p, etc.).	**1. miRNA expression differences:** Compared with CON, 25 miRNAs showed expression differences in the CHO group during the recovery period, with Let7i-5p and miR-195-5p being the most sensitive and specific markers for distinguishing the two groups.<br><br>**2. In vitro mechanism validation:** Overexpression of Let7i-5p and miR-195-5p in C2C12 myoblasts reduced the expression, ubiquitination levels, and proteasome activity of protein degradation-related genes (Foxo1, Trim63, Casp3, Atf4).<br><br>**3. Pathway regulation:** Overexpression of Let7i-5p down-regulated energy sensing and glycolysis genes, and overexpression of miR-195-5p down-regulated lipid metabolism genes.<br><br>**Conclusion:** This study reported that carbohydrate intake during post-exercise recovery was associated with distinct changes in skeletal muscle miRNA expression profiles, occurring in parallel with markers related to protein metabolism and recovery processes.

Author (Year, Country)	Design	Population	Intervention	Cycle and Follow-up	Mechanistic Exploration	Findings and Conclusions
Tang Donghui et al.(2019), China	Quasi-random study	**1. N=67;**<br><br>**2. Traits:** Obese male adolescents aged 12-18 years (BMI ≥23.9 kg/m²); no history of cardiovascular disease, smoking, or hormone use; no regular exercise habits.	**Study Protocol:** 2 groups of random control.<br><br>① Experimental group: Exercise (aerobic + resistance training, 5+2 times weekly) + Nutrition (calorie restriction, balanced nutrients)<br><br>② Control group: Maintain sedentary lifestyle, no exercise or dietary intervention	**1. Intervention duration:** 6 weeks<br><br>**2. Follow-up and Measurement:** Baseline, 6 weeks later.<br><br>**3. Sample:** The measurement indexes were anthropometric indexes, biochemical indexes (glucose and lipid metabolism, NO, ET-1), microRNA-126 expression and reactive congestion index (RHI).	**1. Technique:** Real-time quantitative PCR (qPCR).<br><br>**2. Analytical indicators:** The relative expression level of serum microRNA-126 (miR-126) was analyzed.	**1. Body composition and metabolic improvement:** The body weight, BMI, waist circumference, neck circumference and hip circumference of the experimental group were decreased; the total cholesterol, triglyceride, LDL, blood glucose, insulin and insulin resistance index (HOMA-IR) were improved.<br><br>**2. Endothelial function improvement:** The experimental group showed improved microvascular endothelial function, which was reflected in the increased reactive hyperemia index (RHI) and the ratio of NO/ET-1.<br><br>**3. Epigenetic regulation changes:** The serum miR-126 expression level in the experimental group decreased after intervention. Correlation analysis showed that the change in miR-126 (ΔmiR-126) was positively correlated with the change in BMI (ΔBMI) and RHI (ΔRHI), and negatively correlated with the change in NO/ET-1 (ΔNO/ET-1).<br><br>**Conclusion:** The findings indicated that combined exercise and dietary interventions were associated with changes in serum miR-126 expression, alongside improvements in metabolic parameters and microvascular endothelial function in obese adolescents.
