# Supplementary material for: Exercise and nutrition as epigenetic regulators of gene expression: an exploratory scoping review with bibliometric analysis
Source: Front Nutr. 2026 Mar 10;13:1773920. doi: 10.3389/fnut.2026.1773920 (PMC13008672; doi:10.3389/fnut.2026.1773920)
Supplement: Supplementary file 5 [file Data_Sheet_5.pdf]

Appendix 5

Table S5. Keywords Betweenness Centrality

Node	Betweenness	Closeness	PageRank
exercise	58.284	0.063	0.152
inflammation	12.611	0.048	0.087
dna methylation	12	0.04	0.049
supplementation	6.13	0.045	0.069
oxidative stress	2.159	0.043	0.051
skeletal-muscle	1.816	0.043	0.049
expression	0	0.036	0.019
antioxidant	0	0.037	0.033
athletic performance	0	0.037	0.033
nitric-oxide	0	0.036	0.019
weight-loss	0	0.036	0.019
physical activity	0	0.027	0.021
blood	0	0.032	0.039
responses	0	0.031	0.028
microrna	0	1	0.048
skeletal muscle	0	1	0.048
interval walking training	0	1	0.048
physical-fitness	0	1	0.048
resistance exercise	0	0.5	0.048
ingestion	0	0.5	0.048
protein-synthesis	0	0.5	0.048

Note:The keywords marked in yellow are those with betweenness centrality greater than zero
